# Deciphering Immune Landscape Remodeling Unravels the Underlying Mechanism for Synchronized Muscle and Bone Aging

**DOI:** 10.1002/advs.202304084

**Published:** 2023-12-13

**Authors:** Pengbin Yin, Ming Chen, Man Rao, Yuan Lin, Mingming Zhang, Ren Xu, Xueda Hu, Ruijing Chen, Wei Chai, Xiang Huang, Haikuan Yu, Yao Yao, Yali Zhao, Yi Li, Licheng Zhang, Peifu Tang

**Affiliations:** ^1^ Senior Department of Orthopedics The Fourth Medical Center of PLA General Hospital Beijing 100048 China; ^2^ National Clinical Research Center for Orthopedics Sports Medicine & Rehabilitation Beijing 100048 China; ^3^ Analytical Biosciences Limited Beijing 100191 China; ^4^ The Department of Orthopedic Surgery Second Affiliated Hospital of Harbin Medical University Harbin 150086 China; ^5^ State Key Laboratory of Cellular Stress Biology School of Medicine Faculty of Medicine and Life Sciences Xiamen University Xiamen 361102 China; ^6^ Center for Healthy Aging and Development Studies National School of Development Peking University Beijing 100871 China; ^7^ Central Laboratory Hainan Hospital of Chinese People's Liberation Army General Hospital Sanya 572013 China

**Keywords:** aging, bone loss, muscle loss, sarcopenia, single cell analysis

## Abstract

Evidence from numerous studies has revealed the synchronous progression of aging in bone and muscle; however, little is known about the underlying mechanisms. To this end, human muscles and bones are harvested and the aging‐associated transcriptional dynamics of two tissues in parallel using single‐cell RNA sequencing are surveyed. A subset of lipid‐associated macrophages (triggering receptor expressed on myeloid cells 2, *TREM2* ^+^ Macs) is identified in both aged muscle and bone. Genes responsible for muscle dystrophy and bone loss, such as secreted phosphoprotein 1 (*SPP1)*, are also highly expressed in *TREM2^+^* Macs, suggesting its conserved role in aging‐related features. A common transition toward pro‐inflammatory phenotypes in aged CD4^+^ T cells across tissues is also observed, activated by the nuclear factor kappa B subunit 1 (NFKB1). CD4^+^ T cells in aged muscle experience Th1‐like differentiation, whereas, in bone, a skewing toward Th17 cells is observed. Furthermore, these results highlight that degenerated myocytes produce BAG6‐containing exosomes that can communicate with Th17 cells in the bone through its receptor natural cytotoxicity triggering receptor 3 (NCR3). This communication upregulates CD6 expression in Th17 cells, which then interact with *TREM2* ^+^ Macs through CD6‐ALCAM signaling, ultimately stimulating the transcription of *SPP1* in *TREM2* ^+^ Macs. The negative correlation between serum exosomal BCL2‐associated athanogene 6 (BAG6) levels and bone mineral density further supports its role in mediating muscle and bone synchronization with aging.

## Graphical abstract

This study examines the aging features of the muscle‐bone unit at a single‐cell level. Immune cells are found to play a crucial role in orchestrating the synchronized aging of muscles and bones. The findings advance the understanding of synchronized musculoskeletal aging and lay the molecular and cellular foundation for further basic and translational studies.

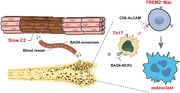

## 1 Introduction

Aging‐related changes in skeletal muscle and bone begin early in life and manifest as a reduction in both the mass and function of these tissues.^[^ ^1^ ^]^ Disorders resulting from such aging‐related changes in either or both tissues have been termed Sarcopenia, Osteoporosis, or osteoSarcopenia (SOS), which significantly constrain the daily activities of elderly people. Patients with SOS are prone to falls and fractures, leading to increased disability, frailty, hospitalization, and mortality, and, along with an aging population, the disease burden of SOS is surging.^[^ ^2^, ^3^ ^]^ Currently, no pharmaceutical intervention is available for sarcopenia.^[^ ^3^ ^]^ Therapeutic agents have long been clinically applied and shown effectiveness for osteoporosis, whereas side effects such as necrosis of the jaw, atypical fractures, etc., are presented.^[^ ^4^ ^]^ Therefore, it remains critical to obtain an in‐depth understanding of the mechanisms driving muscle and bone aging and to identify potential therapeutic targets to prevent the loss of muscle and bone in elderly individuals.

Numerous studies have revealed a synchronization of muscle and bone mass for decades, and a close functional and developmental relationship exists between the two tissues;^[^ ^5^ ^]^ thus, the concept of a muscle‐bone (M&B) unit has emerged.^[^ ^6^ ^]^ Nevertheless, the mechanism underlying the coordination of muscle and bone mass has been previously simplified and viewed as mechanical in nature, where the muscle is recognized as the primary source of anabolic mechanical stimuli for bone tissue.^[^ ^7^ ^]^ Recent findings have identified the endocrine functions of both bone and muscle.^[^ ^8^ ^]^ Therefore, more complex interactions between muscle and bone, in terms of biochemical crosstalk, metabolic communications, and extracellular vesicle‐mediated coupling, are gaining increasing attention.^[^ ^8^ ^]^ Moreover, both tissues consist of diverse cell types at different stages, among which immune cells play an essential role in regulating their homeostasis.^[^ ^9^, ^10^ ^]^ Recent studies have emphasized the impact of conserved immune responses on aging physiology across various organs and shared immune responses may contribute to various aging‐related diseases.^[^ ^11^ ^]^ However, a comprehensive analysis of the cellular composition of human bone and muscle and how these cells adapt and interact during aging is lacking. In this study, we established a single‐cell landscape of human muscle and bone in parallel, depicting the aging features of human M&B units, which could advance the understanding of synchronized musculoskeletal aging and lay the molecular foundation for further translational studies.

## 2 Results

### 2.1 Aging Increases the Diversity of Musculoskeletal Executive Cells and Myeloid Cells

**Figure 1:**
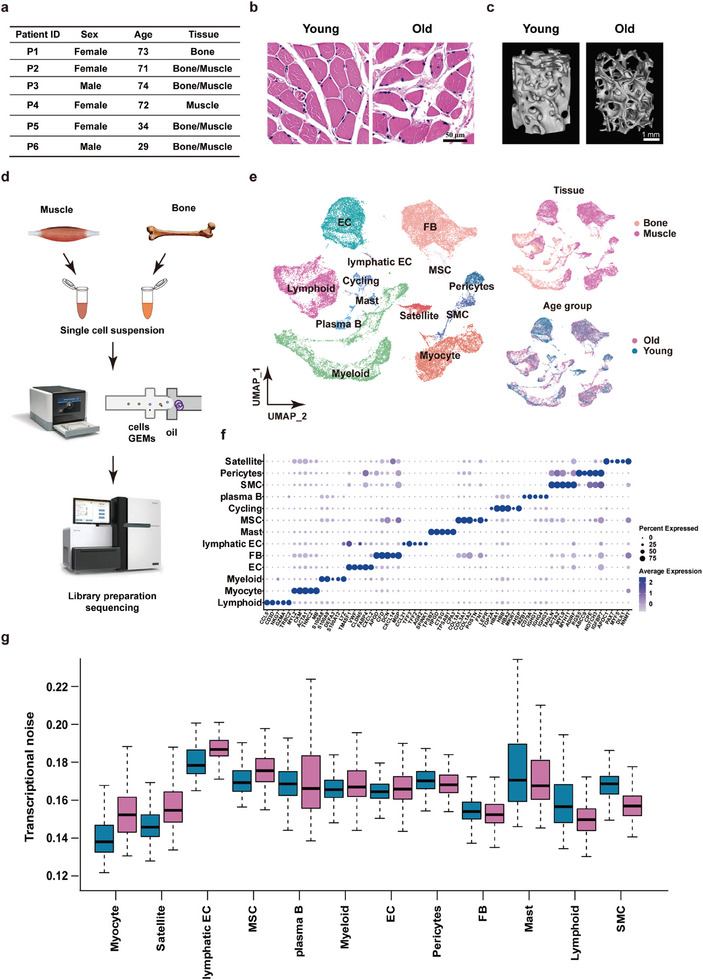
Study design and the cellular landscape of the aging human musculoskeletal system. a) The baseline information of enrolled patients is shown. b) Representative H&E staining images showing a cross‐section view of myofibers from young and older individuals. c) Representative micro‐CT reconstruction images showing the trabecular bones from young and older individuals. d) Schematic representation of our workflow; for enrolled patients, both bone and muscle were dissected. Cells from bone and muscle tissues were isolated, purified, and generated as a single‐cell library separately. e) Cell clustering projected by UMAP plots showing major cell types in musculoskeletal tissues detected by scRNA‐seq; colored by tissue (top) and age distribution (bottom). EC: endothelial cells, FB: fibroblasts. SMC: smooth muscle cells, MSC: mesenchymal stem cells. f) Dot plot showing signature gene expression in each cell type. Circle size indicates the cell fraction expressing the signature gene, and color indicates the gene expression level. g) Transcriptional noise comparison of major cell types between young and old individuals. Blue: young individuals. Red: old individuals.

To explore the cellular diversity and transcriptional adaption of human musculoskeletal tissue during aging, four old (aged over 70 years) and two young (29 and 34 years) individuals were enrolled in this study (**Figure** **1**a). Tissue samples from the gluteus maximus and femoral neck were harvested during hip replacement surgery from each individual. Histologically, we observed that the muscle fiber size was reduced in the elderly group compared to that in the young group (Figure 1b). In addition, we found an increase in extracellular collagen deposition in the interstitial tissues of all older muscles compared to young subjects (Figure 1b). Regarding the bone, micro‐CT reconstruction images showed a decline in trabecular bone number and thickness in older adults compared to that in young adults (Figure 1c). Thereafter, cells in the harvested muscle and bone were isolated, purified, and subjected to droplet‐based single‐cell RNA sequencing (scRNA‐seq) to investigate cell type‐specific remodeling during aging (Figure 1d). After we removed low‐quality or contaminated cells, a total of 40238 cells were obtained, comprising endothelial cells (ECs), fibroblasts (FBs), lymphatic ECs, mesenchymal stem cells (MSCs), pericytes, smooth muscle cells (SMCs), myocytes, satellite cells, myeloid cells, lymphoid cells (mainly T cells and natural killer (NK) cells), plasma B cells, mast cells, and a population of cycling cells (Figure 1e). A UMAP plot showing individual donors’ contributions to the identified cell subsets indicated consistency between donors within the same group (Figure S1a, Supporting Information). By comparing tissue distributions, most cells in the bone were immune cells, while only a few ECs and MSCs were identified. Consistent with the current knowledge,^[12^ ^]^ satellite cells were dominant in young individuals, and an increased lymphoid cell composition was observed in old individuals (Figure 1e; Figure S1b, Supporting Information). The signature expression of each cell type indicated robust filtering and clustering (Figure 1f). Fibrogenic signatures in FBs were minimally expressed in both old and young individuals, suggesting the absence of muscle injury in the enrolled individuals; therefore, we could assume any experimental differences would reflect aging‐driven alterations (Figure S1c, Supporting Information). Furthermore, somatic mutations accumulate during aging; consequently, more noise or transcriptional instability should be observed. Hence, the concept of transcriptional instability was introduced by Enge et al.^[^ ^13^ ^]^ and considered as a feature of mammalian aging,^[13^ ^]^ we calculated the transcriptional noise of the 12 identified cell clusters, six clusters displayed increased noise namely myocytes, satellite cells, lymphatic EC, MSC, myeloid cells, and EC, while the rest 6 clusters were either unchanged or decreased in the old samples (Figure 1g). To understand the underlying mechanisms of differential transcriptional regulation in aging, we subsequently subclustered the major cell types, with a focus on the immune compartment, and delineated their interactions with tissue executive cells in the following sections. Additionally, a landscape of ECs, pericytes, MSCs, and SMCs is also displayed in Figures S1d and S2 (Supporting Information).

### 2.2 A Unique *TREM2^+^* Macrophage Enriched in Aged Musculoskeletal Tissues Highlights a Potential Culprit of Aging‐Related Pathologies

**Figure 2:**
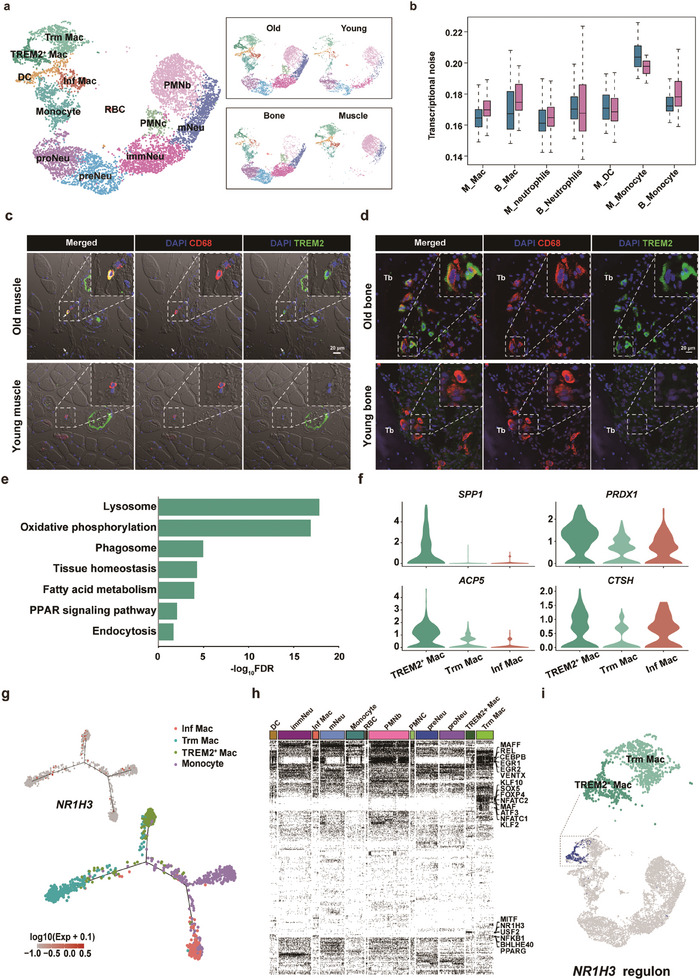
Cellular diversity of myeloid cells in human musculoskeletal tissues. a) UMAP plots of myeloid cell subsets, colored by tissue (bottom) and age distribution (up). b) Transcriptional noise comparison of myeloid cell subsets between young and old individuals. Blue: young individuals. Red: old individuals. M_ = Muscle_; B_ = Bone_. c,d) Representative images of immunofluorescent staining for *TREM2^+^* macrophages (Macs) in both muscle and bone from young and aged individuals. e) KEGG enrichment of highly expressed genes in *TREM2* ^+^ Macs. f) Violin plots of expression levels of representative genes enriched in the GO terms, namely tissue homeostasis in (e). g) Pseudo‐time analysis by Monocle estimating macrophage development in musculoskeletal tissues and pinpointing that *NR1H3* may play a distinct role in the development of *TREM2* ^+^ Macs. h) SCENIC analysis indicated the regulon of *NR1H3* was switched on in *TREM2* ^+^ Macs.Black bars suggested that TF regulon was active in the corresponding cell subsets. i) Transcription factor (TF) in myeloid subsets.

As a central player in innate immunity, we first investigated age‐related changes in the myeloid cell compartments.^[^ ^14^ ^]^ Myeloid cells, including macrophages, monocytes, dendritic cells, and neutrophils, were also identified (**Figure** **2**a). We observed an increased proportion of macrophages among the myeloid cell population in old individuals compared to young individuals (Figure S3a, Supporting Information). Moreover, increased transcriptional noise was also observed in aged macrophages, indicating that aging caused higher variability and potential fate drift in macrophages (Figure 2b). Re‐clustering of macrophages identified three subsets (Figure 2a; Figure S3a,b, Supporting Information): 1) a subset that highly expressed *TREM2*, named *TREM2* ^+^ Macs; 2) a subset that was distinguished by the high expression of the inflammatory marker, *IL‐1B*, named inflammatory (Inf) Macs; and 3) tissue‐resident (Trm) Macs expressing tissue residence markers including *LYVE1* and *MRC1* (Table S1, Supporting Information). By assessing tissue distribution, we found that Trm Macs were exclusively located in skeletal muscle, whereas Inf Macs and *TREM2* ^+^ Macs were found in both tissues (Figure 2a; Figure S3a, Supporting Information). Stratified by age, Trm Macs and Inf Macs were found in both young and aged individuals, whereas *TREM2* ^+^ Macs emerged uniquely in old muscles and bones (Figure 2a; Figure S3a,c, Supporting Information). The transcriptional signature genes of Trm Macs were analyzed using gene ontology (GO) analysis to gain a deeper understanding of their function. As shown in Figure S3d (Supporting Information), genes enriched in Trm Macs were associated with three major function categories: 1) cellular response to multiple niche signals including tumor necrosis factor, insulin stimulus, and interferon‐gamma, and transforming growth factor beta, etc.; 2) muscle homeostasis maintaining, namely angiogenesis, endothelial cell proliferation, aging, skeletal muscle cell differentiation; 3) immune cell‐intrinsic function, namely regulation of apoptotic process and receptor‐mediated endocytosis, positive regulation of T cell activation, antigen processing and presentation of exogenous peptide antigen via MHC class II, and inflammatory response. Similar to our study, prior rodent research also identified *Lyve1* ^+^ muscle resident macrophages, which displayed transcriptional signatures involved in endocytosis, angiogenesis/vascular remodeling, inflammation, and response to chemokines.^[^ ^15^, ^16^, ^17^ ^]^ Regarding Inf Macs, GO analysis revealed an enrichment of signature genes associated with inflammatory processes, including antigen processing and presentation, positive regulation of T cell activation, neutrophil chemotaxis, inflammatory response, and leukocyte cell‐cell adhesion (Figure S3e, Supporting Information). To explore the effects of age‐related changes, we further explored the enriched functions of differentially expressed genes in each macrophage cluster with aging. Being the most prevalent macrophage population in the muscle, Trm Macs in old individuals exhibited enhanced gene expression in regulating leukocyte differentiation, cell chemotaxis, regulation of the ERK1/2 cascade, and exerted a pro‐angiogenic effect (Figure S3f, Supporting Information). Aged Inf Macs displayed features associated with an apoptotic signaling pathway, oxidative stress, positive regulation of NF‐κB transcriptional factor activity, and positive regulation of leukocyte activation (Figure S3g, Supporting Information).

Since *TREM2* ^+^ Macs emerged exclusively in older individuals, we postulated that this subset might show distinguishing features in relation to the aging phenotype of both muscle and bone. We first confirmed the presence of TREM2^+^ Macs in aged muscles and bones by immunofluorescence staining (Figure 2c,d). It has been known that *TREM2* is a key sensor of metabolic pathologies and tissue damage.^18^ ^]^ Prior rodent study has also found that a subset of macrophages with high expression of *Trem2* was chronically activated in the dystrophic muscle of X‐linked muscular dystrophy (MDX) mice, where constant damage occurs.^19^ ^]^ Therefore, the emergence of *TREM2* ^+^ Macs in aged individuals indicates a loss of tissue‐level homeostasis in both muscles and bones. To further understand the functional phenotypes of *TREM2* ^+^ Macs, we explored the functional enrichment of the transcriptional signature genes according to Kyoto Encyclopedia of Genes and Genomes database (KEGG) pathways and GO terms. Results showed that transcriptional signatures of *TREM2* ^+^ macs in muscle and bone were highly associated with lipid metabolism, lysosome, phagocytosis, and tissue homeostasis (Figure 2e). Furthermore, compared to other macrophage subsets, *TREM2* ^+^ Macs also exhibited less inflammatory activation, as indicated by the decrease in the levels of inflammatory response and the upregulation of peroxisome proliferator‐activated receptor (PPAR) signaling and oxidative phosphorylation pathways (Figure S3j, Supporting Information). Such features of *TREM2* ^+^ Macs closely resembled that of adipose tissue tumor‐associated macrophages (TAMs) or brain disease‐associated macrophages (DAMs), which were recently identified in mouse fibrotic lung and fatty liver tissues, as well as in mouse and human adipose and brain tissues, indicating that this cluster may be their musculoskeletal counterpart.^18^ ^]^ Both DAMs and TAMs have been reported to contribute to the pathogenesis of certain tissues by inducing fibrosis (*LGALS3* and *SPP1*) or cellular senescence (*CTSB* and *CD36*). Indeed, genes involved in these biological processes are also highly expressed in *TREM2* ^+^ Macs found in our study (Figure 2f; Figure S3h, Supporting Information). Muscle and bone undergo significant tissue remodeling, including fibrosis, loss of mass, and damage to microarchitecture with aging.^[^ ^2^, ^20^ ^]^ Therefore, the emergence of *TREM2* ^+^ Macs highlights a potential culprit of these aging‐related pathologies. Previous research has also indicated that SPP1 promotes fibrosis in muscular dystrophy ^[^ ^21^ ^]^ and degradation of the bone matrix responsible for bone loss,^22^ ^]^ both of which are recognized as aging phenotypes. Nonetheless, the source of SPP1 in muscle remains unknown; however, SPP1 accumulated in bone was recently believed to originate from macrophages in epididymal adipose tissue through the circulatory system.^[^ ^22^ ^]^ The finding that *TREM2* ^+^ Macs highly expressed *SPP1* in the muscle and bone tissue, which was validated by immunostaining (Figure S3i, Supporting Information), suggests that there is a major source of SPP1 in the local musculoskeletal niche. Accordingly, the identification of *TREM2* ^+^ Macs in aged muscle and bone indicates a loss of tissue homeostasis, and this subset of macrophages may contribute to the pathogenesis of aging‐induced musculoskeletal phenotypes.

To better understand the developmental connection between *TREM2* ^+^ Macs and other macrophages, we constructed pseudo‐time trajectories for the macrophages.^[^ ^23^ ^]^ The in silico trajectory indicated that *TREM2* ^+^ Macs showed distinctive features, as they were distributed at the terminus of the bifurcated branch and showed a minimal connection with Trm Macs, monocytes, and Inf Macs (Figure 2g). Prior rodent experiments have evidenced that the *TREM2* ^+^ Macs counterparts emerged in fatty liver, acne lesions, and tumors originating from recruited circulating monocytes.^[^ ^24^, ^25^ ^]^ Our trajectory also suggested a differentiation potential from monocyte to *TREM2* ^+^ Macs. Along this trajectory, we also revealed that nuclear receptor subfamily 1 group H member 3 (*NR1H3)* was specifically expressed in *TREM2* ^+^ Macs (Figure 2g). Furthermore, gene regulation network analysis using SCENIC identified several transcription factors (TFs), including *MAFF*, *REL*, and *CEBPB*, that modulate macrophage‐specific gene regulatory networks (Figure 2h).^[^ ^26^ ^]^ *NR1H3* was exclusively identified in *TREM2* ^+^ Macs, which agrees with the trajectory inference (Figure 2i). The protein encoded by *NR1H3* belongs to the NR1 subfamily of the nuclear receptor superfamily. NR1 family members are key regulators of macrophage function, controlling the transcriptional programs involved in lipid homeostasis and inflammation.^[^ ^27^ ^]^ Combined with our study results, this suggests a potential role for *NR1H3* in the establishment of a specific transcription program in *TREM2* ^+^ cells.

### 2.3 T Cells Programing Toward Tissue‐Specific Inflammatory Lineages Provides a Mechanistic Basis for Aging‐Associated Chronic Inflammation

**Figure 3:**
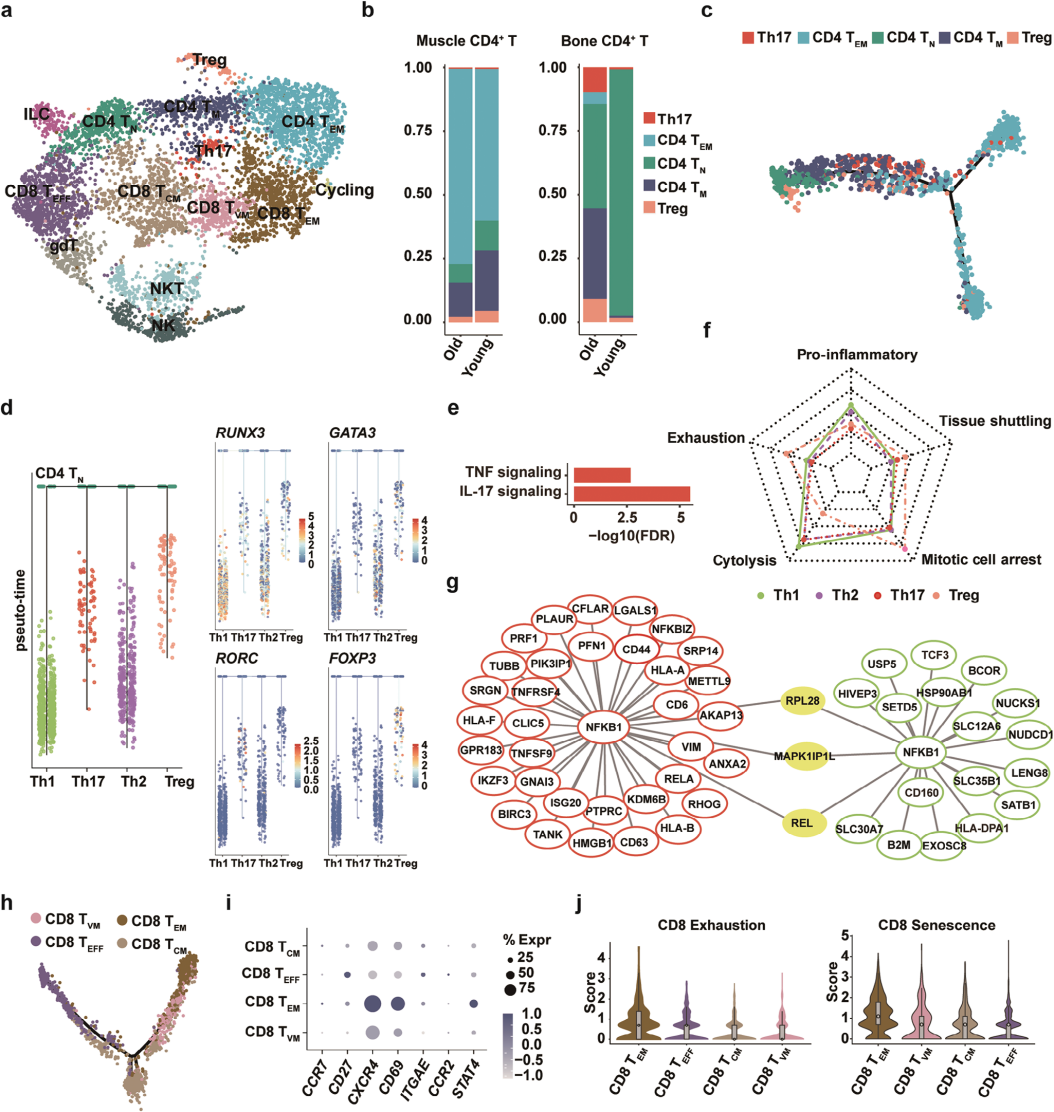
Lymphoid subset reprogramming in aged human musculoskeletal tissues. a) UMAP plots of T and NK cells identified in bone and muscle. b) The proportion of CD4^+^ T cell subsets across age groups, according to tissue type. c) Trajectory inference of CD4^+^ T cells assessed by Monocle. d) Differentiation trajectories of CD4^+^ T cells by URD showing naïve CD4^+^ T cells gave rise to Th1, Th2, Th17, and Treg. The right panel displays the expression of classic transcription factors on each developmental tree. e) GO enrichment for highly expressed genes in Th17. f) The functional enrichment of highly expressed genes in Th1, Th2, Th17, and Treg, visualized by a Radar plot. g) Gene regulatory network inferring NFKB1 targets in Th1 and Th17, respectively. h) Trajectory analysis of CD8^+^ T subsets highlighted that virtual memory CD8^+^ T cells were significantly distinct from effector CD8^+^ T cells. i) Dot plot showing differential gene expression across CD8^+^ T subsets. Circle size indicates the cell fraction expressing the signature gene, and color indicates the gene expression level. j) Exhaustion score and senescence score comparison among CD8^+^ T subsets.

A hallmark of aging is chronic system‐wide inflammation, termed inflammaging. Recent evidence has highlighted intrinsic alterations in T cells actively contribute to inflammaging, and infiltration of these dysfunctional T cells could accelerate tissue‐specific aging phenotypes.^[^ ^28^ ^]^ However, the diversity and age‐associated alterations in T cells infiltrating musculoskeletal tissues are not yet fully understood. Detailed clustering analysis further categorized T and NK cells into 14 subsets (**Figure** **3**a; Figure S4a, Supporting Information), and the percentage of each cell type in both young and old individuals is shown in Figure S4b (Supporting Information). CD4^+^ T cells resided in both muscles and bones, while CD8^+^ T cells were mainly detected in the bone (Figure 3b; Figure S4c, Supporting Information). A bias toward enrichment in memory CD4^+^ T cells and a decrease in the naïve pool was seen both in the muscle and bone during aging (Figure 3b; Figure S4c, Supporting Information), which echoes the notion that aging causes a systemic naïve memory CD4^+^ T cell imbalance.^[^ ^28^ ^]^ In particular, the fraction of effector memory CD4^+^ T (*T*_EM_) cells was elevated in both the muscle and bone, whereas Th17 was almost exclusively detected in bone tissues among old individuals (Figure 3b; Figure S4c, Supporting Information). To gain insight into lineage relationships among these CD4^+^ T cell subsets, we performed a pseudo‐trajectory analysis using a monocle. Naïve T (*T*_N_) cells were diffused at the left end, whereas *T*_EM_ cells were distributed to the right end. A bifurcated distribution was observed in the CD4^+^ *T*_EM_ cells, indicating the existence of two different *T*_EM_ cell phenotypes (Figure 3c). Therefore, we compared the differentially expressed genes between bifurcated *T*_EM_ cells. Prior research has suggested that *T*_EM_ cells maintain effector functionality;^[^ ^29^ ^]^ here, we found that the upper branch *T*_EM_ cell cluster presented a Th1‐lineage transcription pattern (Th1‐like) with high *IFNG* expression, whereas the lower branch mimicked a Th2‐like phenotype (Th2‐like) with *AREG* (Figure S4d, Supporting Information). To confirm our findings, we implemented another trajectory algorithm, URD,^[^ ^30^ ^]^ to delineate the relationships between these T cell clusters. The results showed that Th1‐ and Th2‐like *T*_EM_ cells were both rooted in *T*_N_ cells and distributed separately in independent branches other than Treg or Th17 clusters. In addition, the master regulator genes known for the Th1 (*RUNX3*) and Th2 (*GATA3*) lineages were uniquely highlighted in the corresponding developmental branch (Figure 3d). Therefore, these data indicate that *T*_EM_ cells not only maintain effector functionality but could also be programmed to various effector lineage‐like phenotypes corresponding to the master regulator genes.

Tissue‐specific CD4^+^ T cell programming was observed in aged individuals, as Th1‐ and Th2‐like *T*_EM_ cells were mainly found in aged muscle (Figure S4e, Supporting Information), while Th17 was dominant in aged bone (Figure S4b,c, Supporting Information). We focused on age‐dependent alterations in the function of terminally differentiated CD4^+^ T cells. In agreement with previous studies, Th17 cells were enriched in genes involved in the pro‐inflammatory interleukin‐17 (IL‐17) and tumor necrosis factor (TNF) signaling pathways, both of which are essential for Th17‐mediated tissue inflammation and bone destruction (Figure 3e).^[^ ^31^ ^]^ Th1‐ and Th2‐ like *T*_EM_ cells also exhibited pro‐inflammatory features and strong cytotoxicity (Figure 3f; Figure S4f, Supporting Information),^32^ ^]^ which provided a mechanistic explanation for the inflammation‐induced muscle loss observed in aged muscle.^[^ ^33^ ^]^ Accordingly, these data indicate that a common transition toward pro‐inflammatory phenotypes occurs in aged T cells across tissues. To unravel gene expression regulation beyond such a transition in musculoskeletal aging, we performed gene network analysis by SCENIC for CD4^+^ T cell subsets and discovered that the *NFKB1* regulon was actively transcribed in both aged *T*_EM_ and Th17 cells (Figure S4g, Supporting Information). Furthermore, as the core component of the NF‐κB signaling pathway, *REL* was a direct downstream target in both Th1‐like and Th17 cells (Figure 3g); therefore, we speculated that targeting the NF‐κB signaling pathway may slow down aging phenotypes by attenuating tissue‐level inflammation in both muscle and bone.

Moreover, prior research has identified an accumulation of a small subset of CD8^+^ T cells, termed “virtual memory” CD8^+^ T (*T*_VM_) cells, with aging.^[^ ^34^ ^]^ In mice, *T*_VM_ cells are phenotypically characterized by high expression of *ll2rb* and low expression of *Itga4*. These two markers reflect the semi‐differentiated state of the *T*_VM_ cells. In our sample, a putative *T*_VM_ cell was also identified and showed striking differences from *T*_EM_ cells (Figure 3h). Consistent with a previous study, these cells are naïve‐like and lack the expression of *CCR7* and *CD27* compared to central memory T cells (Figure 3i).^[^ ^35^ ^]^ In our study, *T*_VM_ cells emerged within the bone tissue and increased in proportion with age (Figure S4b,c, Supporting Information). Notably, given that putative *T*_VM_ cells lose the expression of *CCR7*, they may have been included in classical gating strategies for *T*_EM_ cells. Using single‐cell analysis, distinct transcription features were observed between these two subtypes of memory cells. Genes that regulate memory T cell trafficking were less abundant, such as *ITGAE* (Figure 3i),^[^ ^36^ ^]^ while genes that might predict increased responsiveness to the inflammatory milieu (e.g., *STAT4* and *CCR2*) were downregulated in human *T*_VM_ cells (Figure 3i).^[^ ^34^ ^]^ In addition, aged human *T*_VM_ cells exhibited a lower score of exhaustion but a relatively higher score of senescence compared to other memory cells (Figure 3j).^[^ ^37^ ^]^ T cell exhaustion is a state of T cell dysfunction that arises during many chronic conditions and cancer. It is defined by poor effector function, sustained expression of inhibitory receptors, and a transcriptional state distinct from that of functional effector or memory T cells.^[^ ^38^ ^]^ Thus, such results indicate that dysfunctional and senescent *T*_VM_ cells are present in older individuals.

### 2.4 Elevated Blood Interleukin‐18 Levels and Intramuscular LDHA/LDHB Ratios Indicate a Decline in Muscle Function

**Figure 4:**
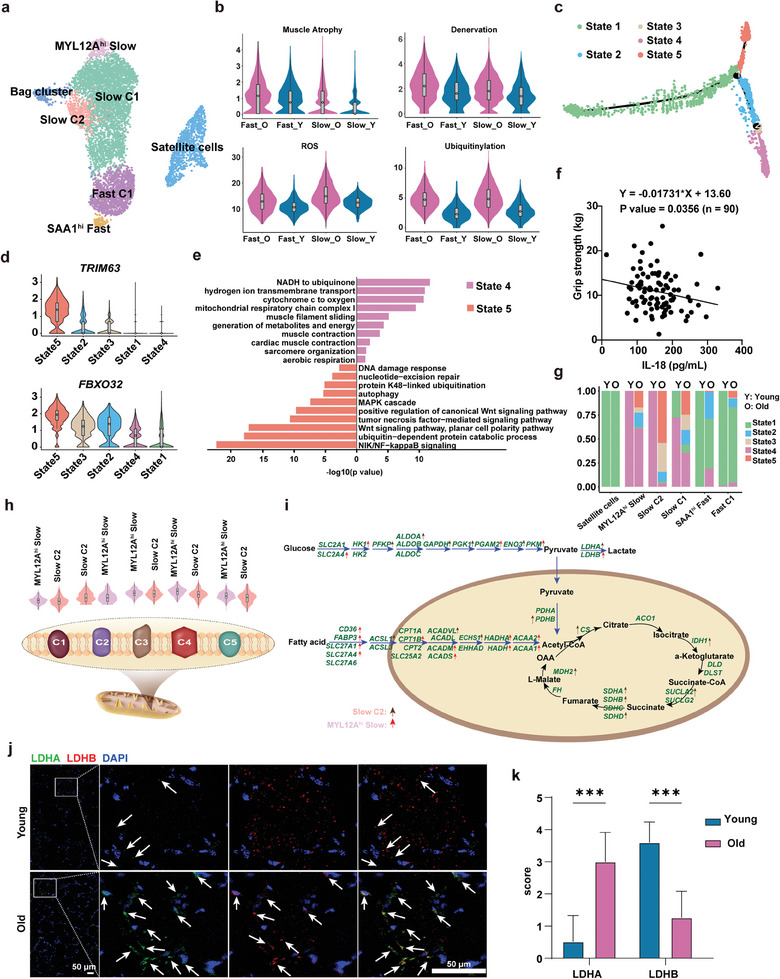
Sub‐clustering analysis of myocytes in aging human muscle. a) UMAP plots showing the diversity of myocyte subsets. b) The functional score of aging‐related terms in fast and slow myocytes between young and old individuals. _O is for _Old; _Y is for _Young. Fast_ is for Fast myocytes; Slow_ is for Slow myocytes. c) The pseudo‐trajectory analysis identified five distinct States in myocytes. d) Violin plot for the expression of muscle‐specific aging hallmark genes (*TRIM63* and *FBXO32*) across the five States. e) GO enrichment of highly expressed genes in State 4 and State 5. f) Pearson's correlation analysis between interleukin‐18 (IL‐18) levels in human serum and grip strength (*n* = 90). g) Comparison of State composition in each subset. h) Overall expression comparison of five mitochondrial respiratory chain complexes in Slow C2 and *MYL12A^hi^* Slow in old individuals. i) Detailed illustration of energy metabolism difference in Slow C2 and *MYL12A^hi^* Slow. The red arrow indicates genes upregulated in the *MYL12A^hi^* cluster, and the grey arrow infers genes upregulated in Slow C2. j,k) Representative Fluorescence in situ hybridization (FISH) images by RNAscope and quantification for LDHA (green) and LDHB (red) expression in young and aged human muscle (*n* = 3). The significance (*p*‐value) was calculated using Pearson's correlation analysis (f) and the Mann–Whitney *t*‐test (k); ^*^ *p* < 0.05; ^**^ *p* < 0.01; ^***^ *p* < 0.001.

Re‐clustering of muscle cells identified six subsets of myocytes and one group of muscle stem cells (MuSCs) (**Figure** **4**a). Among these myocytes, three subsets exhibited slow‐twitched fiber features (highly expressed *MYH7*), including Slow C1, Slow C2, and *MYL12A* ^hi^ Slow; two subsets, namely Fast C1 and SAA1^hi^ Fast displayed fast‐twitched fiber features (highly expressed *MYH1*); and one cluster showed Bag fiber features (highly expressed *MYH15*).^[^ ^39^ ^]^ We calculated scores for aging‐related phenotypes, including muscle atrophy, denervation, ubiquitylation, and reactive oxygen species (ROS) stress. In old individuals, myocytes with fast‐twitch or slow‐twitch phenotypes displayed increased activities regarding these features, suggesting a higher degree of aging (Figure 4b). As hallmark genes for sarcopenia (aging‐related muscle atrophy), *FBXO32* and *TRIM63* were also increased in aged myocytes (Figure S5a,b, Supporting Information).^[^ ^40^ ^]^ We applied trajectory analysis to delineate the relationships between these muscle cells, and subsequently, five functional states were identified (Figure 4c). MuSCs and the majority of cells from young individuals were enriched in State 1, whereas States 2–5 were dominantly occupied by aged myocytes (Figure S5c, Supporting Information). Intriguingly, State 5 was exclusively distributed on one branch of the trajectory while States 2–4 were diffused on the other, which suggested that myocytes of State 5 may present a unique transcript feature. We further examined the functional enrichment score of aging‐related phenotypes and expression levels of sarcopenia markers across each state. State 5 exhibited the highest score for aging phenotypes and expression levels of sarcopenia markers, indicating that myocytes in this State displayed the highest degree of aging (Figure 4d; Figure S5d, Supporting Information). Consistent with this finding, biological processes associated with aging were also enriched in State 5, including DNA damage response, ubiquitination, and autophagy (Figure 4e; Figure S5e, Supporting Information). Differential expression analysis identified that *IL18* was highly expressed in State 5, which encodes a secreted protein, interleukin‐18 (IL‐18) (Figure S5f, Supporting Information). To test whether the expression level of IL‐18 correlated with muscle function, we measured IL‐18 levels in the blood serum of 90 subjects for whom grip strength was evaluated. IL‐18 was negatively correlated with grip strength (R = −0.01731, P = 0.035), suggesting that IL‐18 could be a potential biomarker for the evaluation of muscle function (Figure 4f).

Compared to fast fibers, slow fibers display higher resistance to aging‐associated mass loss according to histological analysis, but the molecular alterations remain unknown at the transcriptome level.^[^ ^41^ ^]^ As a result, we focused on Slow C1, Slow C2, and *MYL12A* ^hi^ Slow, which exhibited slow fiber features. Among these, Slow C1 was present in both young and aged individuals, whereas the other two were exclusively present in aged individuals (Figure S5g, Supporting Information). Thus, we hypothesized that the Slow C2 and *MYL12A* ^hi^ Slow might degenerate from normal slow fibers to various extents. Details of signature genes for Slow C2 and *MYL12A* ^hi^ Slow are listed in Tables S2 and S3 (Supporting Information). To confirm our hypothesis, we first compared the proportion of trajectory State 5 in the two subsets, where State 5 was preferentially distributed in Slow C2 rather than *MYL12A* ^hi^ Slow, indicating that Slow C2 may exhibit a more deteriorated function (Figure 4g). Decreased mitochondrial function and content are indicators of aging in slow fibers.^[^ ^33^ ^]^ Compared to *MYL12A* ^hi^ Slow, Slow C2 had a decreased overall mitochondrial function (Figure 4h). Moreover, aging‐related phenotypes were more prominent in Slow C2 than in *MYL12A* ^hi^ Slow (Figure S5h, Supporting Information). These data are in accordance with our deductions based on the proportion of State 5. Additionally, we characterized the genes involved in fatty acid (FA) β‐oxidation and glucose metabolism in Slow C2 and *MYL12A* ^hi^ Slow. The results indicated that *MYL12A* ^hi^ Slow preserved higher expression of genes mediating FA uptake (*CD36, FABP3*, *SLC27A1*, and *SLC27A4*), transport to the mitochondria (*CPT1B*), and β‐oxidation, which suggested active FA metabolism (Figure 4i). Notably, prior research using bulk sequencing and proteomics has suggested that *FABP3* is upregulated in aged skeletal muscles, causing endoplasmic reticulum (ER) stress, and disrupting muscle homeostasis.^[^ ^42^ ^]^ We found that *MYL12A* ^hi^ Slow may be capable of adapting to this change by increasing the transport of FAs to the mitochondria and β‐oxidation. In contrast, Slow C2 showed aging‐prone *LDHA* expression, whereas *LDHB* was more prominent in *MYL12A* ^hi^ Slow (Figure 4i). This was further confirmed by in situ hybridization of *LDHA* and *LDHB* mRNA (Figure 4j,k; Figure S5i, Supporting Information). Prior research in the brain has shown that a high lactate level is a hallmark of aging and is caused by an increase in the *LDHA/LDHB* ratio.^[^ ^43^ ^]^ Given our evidence of Slow C2 as more aging myocytes, an increase in the *LDHA/LDHB* ratio might be a predictive value for muscle aging phenotypes.

### 2.5 Exosome‐Driven Long‐Distance Cell‐Cell Communication Facilitates Muscle‐Bone‐Synchronized Aging

**Figure 5:**
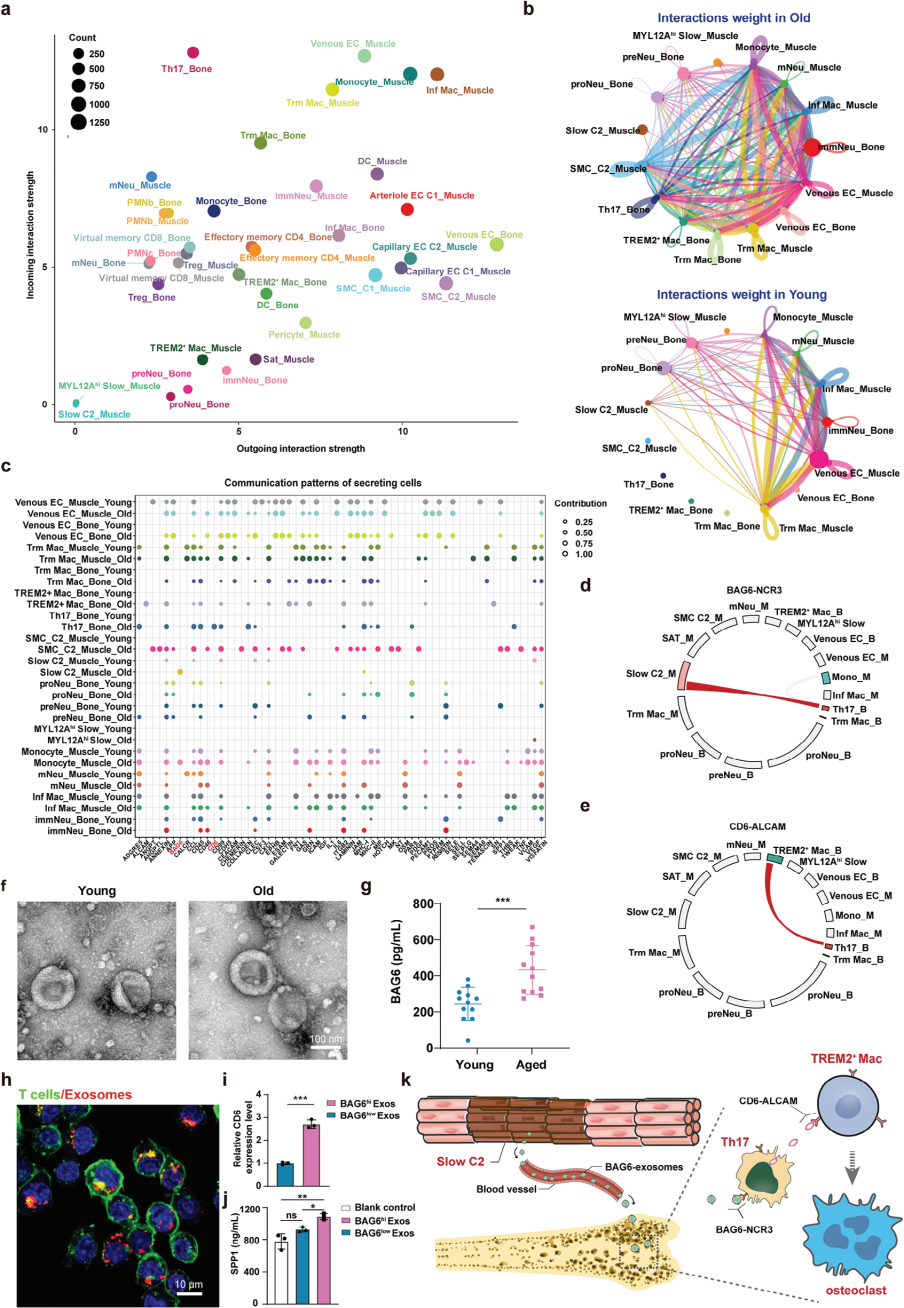
Cellular communication among different cell subsets in human bone and muscle tissues. a) Incoming and outgoing interaction strength of different cell subsets in old individuals. b) Autocrine and paracrine counts of selected cell subsets in old (top) and young (bottom) individuals. c) Cellular communication signaling variation in different cell subsets between old and young individuals. d,e) Circos plot showing the expression of ligand‐receptor pairs *BAG3‐NCR3* and *CD6‐ALCAM*. f) Characterization of exosomes by TEM harvested from the blood plasma of young and old individuals. M for _Muscle, _B for _Bone. g) BAG6 expression in exosomes from human participants in either young or aged groups was determined by enzyme‐linked immunosorbent assay (ELISA)(*n* = 24). h) Representative immunofluorescent images showing the uptake of exosomes by Th cells. Exosomes were labeled by PKH26 and incubated with Th cells for 12 h. i) Intracellular CD6 expression of T cells after incubation with BAG6‐shuttled exosomes compared to PBS controls, as tested by qPCR (*n* = 3 independent experiments). j) Expression level of SPP1 in the conditioned medium of macrophages and Th17 cells co‐culture system, determined by ELSIA. The conditioned medium contained the same quantity of either BAG6^hi^ exosomes (BAG6^hi^ Exos) or BAG6^low^ exosomes (BAG6^low^ Exos), or the same volume of PBS. k) Schematic representation of predicted cell‐cell interactions across aged muscle and bone. Degenerated muscle secretes exosomes containing BAG6, the latter of which travel through blood vessels and promote the interaction between Th17 cells and *TREM2^+^* Macs in bone. The *TREM2^+^* Macs potentially facilitate the differentiation of osteoclasts and bone resorption. The significance (*p*‐value) was calculated using the Mann–Whitney *t*‐test (g and i) and the one‐way ANOVA test (j); ^*^ *p* < 0.05; ^**^ *p* < 0.01; ^***^ *p* < 0.001.

To rewire cell‐cell interactions during aging, CellChat^[^ ^44^ ^]^ was used to construct a comprehensive signaling interaction map between all identified subsets in young and old individuals separately. We then calculated the differential interaction weight between young and old individuals and found that old‐enriched cell subsets had extensively increased crosstalk (Figure S6a, Supporting Information). To further dissect the cellular crosstalk that emerged in old individuals, we investigated communication signals for old‐enriched cell subsets in the aged group (Figure **5**a). We focused on the cell subsets with significantly altered communication. Interestingly, we found that Slow C2 communicated with Th17 in the bone in the aged group (Figure 5b). To further examine the exact ligand‐receptor molecules that participate in the aging network, we delineated the communication signaling for these aging‐related cells. Surprisingly, we identified Slow C2‐secreted *BAG6* signaling molecules exclusively in old individuals (Figure 5c). BAG6 signaling functioned in a paracrine manner and was secreted by Slow C2 and monocytes in the muscle (Figure 5d; Figure S6b, Supporting Information). We focused on the BAG6 from Slow C2 and hypothesized that Slow C2 may interacted with Th17 via the *BAG6‐NCR3* axis (Figure S6b,c, Supporting Information). Prior research has shown that *BAG6*‐encoded protein, also known as BAG6, is released extracellularly via exosomes.^[^ ^45^ ^]^ As Slow C2 in muscle and Th17 in bone were not physically juxtaposed, we assumed that BAG6 might be secreted by aged and malfunctioned muscle via exosomes and thereafter delivered to Th17 through blood vessels. As mentioned above, Th17 cells are known for age‐related bone loss; therefore, to understand the impact of BAG6 on Th17 cells, we further dissected the increased crosstalk between Th17 and other cell clusters in aged bones. Consequently, the *CD6* signaling pathway was of particular interest, as it was enriched in Th17 cells (Figure 5c). Furthermore, *ALCAM* was the only expressed receptor for *CD6*, which was detected in *TREM2* ^+^ Macs in the bone (Figure 5e; Figure S6d,e, Supporting Information). In addition, we applied another algorithm, CellPhoneDB, to compare cell interactions during aging and thereafter validated the increased *BAG6* and *CD6* signaling in silico (Tables S4 and S5, Supporting Information). The aforementioned results indicated that *TREM2* ^+^ Macs were the major macrophage population in aged bone and highly expressed genes that promote osteoclastogenesis and bone destruction, such as *SPP1*.^[^ ^46^ ^]^ To this end, we applied NicheNet^[^ ^47^ ^]^ to predict the downstream targets of *TREM2* ^+^ Macs upon *CD6* stimulation. We found that the transcription factor *STAT3* was significantly upregulated in *TREM2* ^+^ Macs, which could bind to the promoter region of *SPP1* and enhance its transcription (Figure S6f, Supporting Information).^[^ ^48^, ^49^ ^]^

To confirm the in silico findings, we performed validation experiments. First, we assessed BAG6 expression in human muscle and blood exosomes from both aged and young subjects. As expected, the expression of *BAG6* was higher in aged skeletal muscles than in young skeletal muscles (Figure S6g, Supporting Information). Blood plasma exosomes were isolated from the peripheral blood of the included subjects using size‐exclusion chromatography (SEC) and characterized using Transmission electric microscope (TEM), nanoparticle tracking analysis (NTA), and western blotting (Figure 5f; Figure S6h,i, Supporting Information), in accordance with the guidelines recommended by the International Society for Extracellular Vesicles (ISEV). Thereafter, higher expression of BAG6 was found in exosomes derived from aged and osteoporotic participants (Figure 5g; Figure S6j, Supporting Information). To test the effects of BAG6 on Th17 cells, we stimulated human Th17 cells using blood exosomes with higher expression of BAG6 (BAG6^hi^ Exos) from aged individuals and those with lower expression in young individuals (BAG6^low^ Exos) (Figure 5h). In line with the bioinformatic analysis, increased expression of *CD6* was detected in Th17 cells from the BAG6^hi^ Exos group after treatment compared to those from the BAG6^low^ Exos group (Figure 5i). The sorted Th17 cells from old individuals also showed an increased *CD6* expression, as compared to those from young ones (Figure S6k, Supporting Information). Additionally, to determine whether such interactions could ultimately promote the production of *SPP1* in macrophages, we co‐cultured human macrophages with Th17 cells in a conditioned medium with either BAG6^hi^ Exos or BAG6^low^ Exos. Higher expression of the *SPP1*‐encoded protein osteopontin was detected by ELISA in the BAG6^hi^ Exos‐treated group (Figure 5j). We further treated Th17 cells and macrophages with BAG6^hi^ Exo, respectively. Results showed that BAG6^hi^ Exos did not directly stimulate the *SPP1* expression in either macrophages or Th17 cells separately (Figure S6l, Supporting Information). Accordingly, these results support our findings from the bioinformatic analysis, where BAG6 is secreted by aged and malfunctioning muscle via exosomes and thereafter delivered to Th17 cells. This communication upregulates CD6 expression in Th17 cells and ultimately stimulates the production of SPP1 in macrophages, which is responsible for osteoclastogenesis and bone loss. Such long‐distance cell‐cell interactions facilitate synchronized aging within the M&B unit (Figure 5k).

## 3 Discussion

In this study, we established a single‐cell landscape showing heterogeneous aging features of diverse cells within the M&B unit. Aging increased the diversity of myeloid cells, which was indicated by higher levels of transcriptional noise. A distinct subset of macrophages, *TREM2* ^+^ Macs, was identified in aged muscles and bones. TREM2, a transmembrane receptor, interacts with a wide array of ligands that are hallmarks of tissue damage resulting from the loss of metabolic homeostasis and a chronic low‐grade inflammatory state.^[^ ^18^ ^]^ Macrophages highly expressing TREM2 are found in pathological sites across multiple organs, including the fatty liver,^[^ ^24^ ^]^ obese adipose tissue,^[^ ^50^ ^]^ and Alzheimer's brain.^[^ ^51^ ^]^ *TREM2* ^+^ Macs identified in aged muscle and bone shared similar characteristics with these macrophages, implying similar stresses induced by aging within the muscle and bone compartments. Indeed, an increase in fat infiltration has been recognized as a shared metabolic aberration seen in both muscle and bone during aging.^[^ ^52^, ^53^ ^]^ In addition, a systemic low‐grade inflammatory state has been observed as a common feature of aging.^[^ ^54^ ^]^ The findings of this study have broadened our understanding that the emergence of *TREM2* ^+^ Macs may represent a conserved macrophage alteration within the bone and muscle compartments in response to aging‐induced homeostasis aberrations.

Regarding the functions of *TREM2* ^+^ Macs in aged individuals, we found that *SPP1*, which promotes fibrosis in muscular dystrophy and bone destruction, was highly expressed in *TREM2* ^+^ Macs. Similarly, in mice with metabolic‐associated fatty liver disease, *Trem2* ^+^ Macs were found in the fibrotic zone and expressed genes involved in fibrosis, such as *Spp1* and *Des*, suggesting a role in the mechanism of steatohepatitis.^[^ ^24^ ^]^ Consistent with our findings, blockade of *TREM2* inhibited bone resorption, and TREM2 stimulation enhanced the formation of mature osteoclasts that were generated from bone marrow macrophages or RAW264.7 cells treated with receptor activator for nuclear factor κ B Ligand (RANKL) and macrophage colony‐stimulating factor 1 (M‐CSF1). Furthermore, loss‐of‐function mutations of *TREM2* are seen in the context of Nasu‐Hakola disease (NHD), a human disease characterized by osteoporotic features leading to recurrent bone fractures associated with pre‐senile dementia.^[^ ^55^ ^]^ Patients with NHD present osseous symptoms typically at a young age (between 20 and 30 years), where impairment in *TREM2* ^+^ signaling may disrupt the coupling of bone resorption and formation, leading to low turnover osteoporosis. A lack of data comparing aged subjects with and without NHD impedes the understanding of the role of *TREM2* signaling in bone homeostasis. However, the high expression of *SPP1* in *TREM2* ^+^ Macs suggests that this subset of macrophages is a potential culprit in aging‐related pathologies. Given the importance of *TREM2* ^+^ Macs in various diseases, including obesity, Alzheimer's disease, and cancer, numerous efforts are underway to pharmacologically target *TREM2* signaling.^[^ ^18^ ^]^ Our study highlights a novel possibility that harnessing the *TREM2* function might have beneficial effects on the aging phenotypes of M&B units beyond the aforementioned contexts.

Subsequently, we found that T cells residing in aged muscles and bones exhibit pro‐inflammatory features. Most older individuals develop inflammaging, a condition characterized by elevated levels of inflammatory blood markers, which increases the risk of high chronic morbidity, disability, frailty, and premature death.^[^ ^54^ ^]^ Although inflammaging was initially attributed to the accumulation of non‐immune senescent cells, recent evidence has highlighted T cells as major drivers of this age‐associated inflammation.^[^ ^56^ ^]^ However, the effects of aging on T lymphocytes in humans are largely limited to the peripheral blood, which has been more extensively studied than tissue‐residing T cells.^[28^ ^]^ In our study, biased differentiation of CD4^+^ T cells was seen in bone and muscle during aging, where Th1‐like cells were mainly enriched in muscle while Th17 was enriched in bone. Th1 has been previously described to contribute to aging‐related obesity and insulin resistance,^[^ ^57^ ^]^ while Th17 contributes to bone loss.^[^ ^10^ ^]^ Such biased differentiation echoes the aging phenotypes of host tissues. Attempts have been made to modulate certain inflammatory markers to prevent aging‐associated conditions; however, clinical trials have shown controversial results. According to our findings, immune cells may adapt to various phenotypes shaped by the tissue‐specific niche; therefore, a deeper understanding of the immune cell diversity and its role in tissue‐level inflammation is needed to facilitate a more precise modulation. Furthermore, we identified that the NFKB1 regulon is actively transcribed in Th1‐like and Th17 cells, which are the core components of the NF‐κB signaling pathway. NF‐κB is a pleiotropic transcription factor that has been reported to be closely associated with aging‐related dysfunction in various contexts.^[^ ^58^, ^59^ ^]^ In the context of muscle and bone, a new perspective on the role of NF‐κB as a master regulator—coordinating the aging features of M&B units—has been provided here in our study. Nonetheless, the downstream regulons of NF‐κB in Th1 and Th17 were quite different, which further confirmed that T cells were shaped by the local niche and, therefore, operate in a variety of specialized functions.

Muscle has long been recognized as the driving force for bone adaptation through mechanical loading.^[^ ^60^, ^61^ ^]^ Recent evidence has identified muscle as an endocrine organ.^[^ ^62^ ^]^ Therefore, beyond the conserved hallmarks of aging, we wondered if there are certain factors released from aged muscle to facilitate the synchronized aging of the two tissues. Prior research has suggested that fibers with a slow‐twitch phenotype are more resistant to aging‐induced mass loss.^[^ ^63^ ^]^ By analyzing these cells at a single‐cell resolution, we found that the function of slow fibers may also be damaged to various extents, as indicated by the metabolism switch, and secreted factors that are closely correlated with grip strength. In addition, we identified that exosome‐shuttled BAG6 might play a crucial role in mediating muscle and bone coupling, as BAG6 is highly expressed in muscles with a higher degree of aging and interacts with Th17 in bone through the *BAG6*‐*NCR3* axis. Moreover, increased expression of exosomal BAG6 was observed in patients with osteoporosis compared with that in normal subjects. Therefore, beyond soluble factors, including irisin^[^ ^64^ ^]^ and myostatin,^[65^ ^]^ our findings may provide a new perspective that muscle and bone could interact through exosome‐mediated signaling.

## 4 Experimental Section

### Human Participants

This study was approved by the Ethics Committee of the Chinese PLA General Hospital, and written informed consent was obtained from all patients. All experiments were performed in accordance with approved protocols. Samples, including muscle, bone, and blood plasma, were harvested from patients recruited from the Chinese PLA General Hospital hip fracture cohort.^[^ ^66^ ^]^ Ninety human serum samples were used to examine the relationship between serum IL‐18 levels and grip strength. The samples were randomly selected from the China Hainan Centenarian Cohort Study (CHCCS).^[^ ^67^ ^]^

### Hematoxylin and Eosin (H&E) Staining of Muscle Tissue

Human muscle tissues were fixed with 4% paraformaldehyde for 24 h and dehydrated and embedded in paraffin. Five micrometers thick muscle slides were obtained and stained with HE according to the manufacturer's instructions. In brief, tissue sections were dewaxed and dehydrated, and then incubated for 10 min with hematoxylin solution and washed with water. Eosin was added for 1 min and sections were dehydrated with ethanol. Sections were captured using bright field microscopy (Leica DM IL LED).

### Microcomputed Tomography (CT) Analyses

The micro‐CT scanning of bone specimens was performed using the Inveon MM system.^[^ ^68^ ^]^ The exposure time was set to 1500 ms in each of the 360 rotational steps with an effective pixel size of 8.89 µm, a voltage of 60 kV, and a current of 220 µA. The images have 1536 slices, and the voxel size was 8.89 µm in all three axes. The 2D images were then reconstructed with Inveon Research Workplace to create 3D visualization images. A circle with a diameter of 3.71 mm on consecutive trans‐axial sections (with a height of 4.4 mm) was used to create a cylindrical volume of interest.

### Tissue Dissociation and Cell Isolation

Bone and skeletal muscle were obtained from patients who underwent hip replacement surgery. For skeletal muscle, tissues were dissociated by enzymatic digestion with a skeletal muscle dissociation kit according to the manufacturer's instructions. The bone tissue was cut into small pieces and digested with trypsin for 10 min at 37 °C and followed by type II collagenase overnight at 37 °C. Enzymatic digestion was stopped with DMEM supplemented with 10% fetal bovine serum (FBS). The cell pellet was collected by centrifugation at 300 × g for 5 min, resuspended in red blood cell (RBC) lysis buffer, and incubated on ice for 2 min. To remove cell debris and dead cells, RBC‐lysed cells were further purified using the Dead Cell Removal Kit. Finally, the isolated cells were passed through a 70 mm filter and subjected to a single‐cell experiment.

### 10x Genomics 3 Single‐Cell RNA‐Seq

The single‐cell suspension was processed through the Chromium Single‐Cell platform using the Chromium Single‐Cell 3′ Library and Gel Bead Kit v3. Briefly, ≈15 000 individual cells were loaded onto the Chromium Single‐Cell A Chip Kit and partitioned into gel beads in emulsion in the chromium instrument, where the cells and barcoded reverse transcription occurred, followed by amplification, fragmentation, and 5′ adaptor and sample index attachment. Libraries were sequenced using an Illumina Nova‐seq system.

### Computational Analysis of scRNA‐Seq Data

Sequencing reads were aligned to the GRCh38 human genome using the CellRanger toolkit with default parameters. Low‐quality cells were discarded according to the following criteria: 1) cells that had fewer than 400 genes, 2) cells that had fewer than 600 UMI or over 10000 UMI, and 3) cells that had more than 15% mitochondrial UMI counts. After quality control, SCTranform wrapped in the Seurat package was applied to integrate the expression matrices of each sample and remove the batch effects of donors. The integrated matrix was analyzed (dimension reduction, graph‐based clustering, marker gene detection, and visualization) using Seurat software. Genes presented in fewer than ten cells were filtered out (UMI > 0). Briefly, highly variable genes (HVGs) were calculated with the “FindVariableFeatures,” and the top 3000 HVGs were selected for downstream analysis. Data were scaled using the “ScaleData” function, setting the parameters “vars.to.regress” to “percent. mito” and “nUMI.” Principal component analysis (PCA) was performed using the “RunPCA” function with the top 3000 HVGs. The number of principal components (PCs) was selected using a visualization plot with the “ElbowPlot” function. A shared nearest neighbor (SNN) graph was constructed using the “FindNeighbors” function with the top 40 PCs, and the cells were clustered by the “FindClusters” function with the “resolution” parameter set to 0.5. The “RunUMAP” function was used for the visualization plot. Marker genes for each cluster were detected using the “FindAllMarkers” function, setting the parameter “min. pct” to 0.2 and “logfc. threshold” to 0.4.

For myeloid, TNK, myocytes, smooth muscle cells/pericytes, and endothelial cell populations, the number of PCs used in the “FindNeighbors” function was 30, 30, 15, 30, and 30, respectively, while the “resolution” parameter of the “FindClusters” function was set to 0.5 for all five populations. Subsequently, cell clusters were manually annotated to the major cell types according to known markers, and any cluster with multiple markers for the two cell types was manually discarded as a doublet. Functional enrichment for certain cell subsets was performed using the clusterProfileR package with differentially expressed genes.

### Gene Set Score Analysis

Signature genes were retrieved for Reactive oxygen species stress (ROS) and denervation from the Gene Ontology (GO) database (GO:1 903 409 and GO:00 14894, respectively). Muscle atrophy was calculated using the mean expression values of *TRIM63, RPS6KB1*, *PPARGC1A*, *CFLAR*, *MSTN*, *MYOG*, and *ACTN3* according to the GO database (GO:00 14889). The genes involved in ubiquitin were calculated using the mean expression values of *TRIM63*, *TRIM54*, *TRIM32*, *FBXO30*, *NEDD4*, *TRAF6*, *FBXO32*, *CBLB*, *KLHL40*, *KBTBD13*, *KLHL41*, and *KLHL20*.^[^ ^69^ ^]^ Exhaustion scores for the identified T cell subsets were obtained by calculating the average expression of exhaustion genes including *LAG3*, *TIGIT*, *PDCD1*, *HAVCR2*, and *CTLA4*.^[^ ^70^ ^]^ The T‐cell senescence score was calculated using the mean expression values of *TP53*, *CDKN2A*, *TOP1*, *TP63*, *LMNB1*, *CDKN1A*, *MKI67*, and *CD28*.^[^ ^71^, ^72^ ^]^ The signature scores were computed by the Seurat function “AddModuleScore”.

### Pseudo‐Trajectory Analysis of Myeloid Cells, T Cells, and Myocytes

The monocle package (version 2.12.0) was used to infer the potential pseudo‐times for myeloid cells, CD8^+^ T cells, and myocytes. After size factor calculation and dispersions estimation, differentially expressed genes among clusters along the trajectory were identified by the “differentialGeneTest” function. The *q* values were set to 10E‐15, 10E‐35, 10E‐10, and 10E‐25 to determine the significance of macrophages, neutrophils, CD4^+^ T cells, and myocytes, respectively. For CD8^+^ T cells, the top 1000 genes ordered by *q* value were selected. Dimension reduction was performed using the “reduceDimension” function with the “DDRTree” method. After cell ordering, the “plot_cell_trajectory” function was used for visualization.

To better understand the developmental connections among CD4^+^ T cells, another algorithm, URD (version 1.0.1),^[^ ^30^ ^]^ was implemented to reconstruct the trajectory. Naïve CD4^+^ T cells were set as the root cells, and Th17, Treg, and monocle‐defined Th1/Th2 cells were included. The following parameters were adopted in the study: variable genes were defined as diffCV.cutoff = 0.7, knn = 200, sigma = 8, the k‐divergence method, and 100 for cells.per.pseudotime.bin.

### Gene Expression Regulatory Analysis

To infer the gene regulatory network, the single‐cell regulatory network inference and clustering (SCENIC)^[^ ^26^ ^]^ algorithm was used to identify regulons specifically involved in different cell subsets. The raw expression matrix was extracted, and the transcription factor (TF) activities (AUCell) for each cell were calculated using motif collections version mc9nr. The significantly upregulated regulon was defined by a log fold change of more than 0.1 and an adjusted *p*‐value < 10E‐5. The transcriptional network of TF and predicted target genes were visualized using Cytoscape and the igraph package.

### Cell‐Cell Interaction Analysis

To investigate cellular communication in multiple dimensions, three algorithms were adopted. First, the SCtranformed data were used as input for CellphoneDB,^[^ ^73^ ^]^ and the parameter “iterations” was set to 1000, “threshold” to 0.1, and “*p*‐value” to 0.05. The outgoing and incoming signals were inferred by the CellChat package.^[^ ^44^ ^]^ Cellular communications found in more than ten cells for downstream analysis were maintained. After selecting candidate ligand‐receptor pairs, NicheNet^[^ ^47^ ^]^ was applied to further predict the target genes in the receiver cells for a specific ligand. Upon ligand CD6 stimulation, the predicted downstream target genes in *TREM2* ^+^ Macs were visualized using Cytoscape.

### Quantification of Serum Interleukin 18 in the Blood

A simple Plex assay was performed using the Ella System to determine the protein concentration of IL‐18 in serum, according to the manufacturer's instructions. Briefly, 50 µL of diluted sample was loaded into separate wells of the cartridge, and 2 mL of washing buffer was loaded into the respective wells. The assay was then run using Simple Plex Runner Software and analyzed using Simple Plex Explorer.

### RNAscope Assay for *LDHA* and *LDHB* mRNA Detection

RNAscope 2.0 Assays were performed using the RNAscope Multiplex Fluorescent Reagent Kit v2 according to the manufacturer's instructions. Hs*‐LDHA*, Hs‐*LDHB*‐C2 targeting label probes, and control probes were ordered from the ACD. After counterstaining and mounting the slides, all images were captured using a confocal laser scanning microscope. Staining data were recorded according to the semiquantitative guidelines provided by ACD: 0 for no staining or < 1 dot/10 cells, 1 for 1–3 dots/cell, 2 for 4–9 dots/cell, and none or very few dot clusters, 3 for 10–15 dots/cell and < 10% of the dots in clusters, and 4 for > 15 dots/cell and > 10% of the dots in clusters.

### Immunostaining for *TREM2^+^* Macs in Muscle and Bone

The muscle samples were fixed in 4% paraformaldehyde (PFA) for 24 h, dehydrated and embedded in paraffin, and finally sectioned to obtain 4 µm‐thick paraffin‐embedded muscle sections. Following deparaffinization and dehydration, the slides were immersed in citrate buffer and boiled for 10 min for antigen retrieval. After blocking with QuickBlock blocking buffer at room temperature for 30 min, the muscle sections were incubated overnight at 4 °C with primary antibodies against CD68 (1:500) and TREM2 (1:250). After washing three times, the sections were incubated for 1 h at room temperature with the secondary antibodies Alexa Fluor 488 (1:500) and Alexa Fluor 568 (1:500). After counterstaining and mounting the slides, all images were captured using a confocal laser scanning microscope (Leica TCS SP5, Germany). For bone samples, cryosection was used for immunostaining following the established protocols.

### Exosome Isolation and Characterization

Exosomes were isolated from the plasma of both young and aged individuals using size‐exclusion chromatography (qEV single 35 nm iZON columns) according to the manufacturer's instructions. The exosomes collected from 6 to 11 fractions were characterized by nanoparticle tracking analysis using the NanoSight NS300. The morphology of the isolated exosomes was characterized using transmission electron microscopy (TEM). Exosomes were then characterized for ALIX, CD9, TSG101, CD63, and CD81 markers using western blotting, according to a previously described protocol.

### Enzyme‐Linked Immunosorbent Assay (ELISA) for BAG6 Detection

BAG6 levels were measured using the Human HLA‐B‐associated transcript 3 (BAT3) ELISA kit according to the manufacturer's protocol and based on the double antibody sandwich technique.

### Exosome Uptake and Confocal Microscopy

The exosomes extracted by the size exclusion method were incubated with PKH26 at a ratio of 1:200 at 4 °C overnight. The exosomes were washed three times with PBS using 30 kDa ultrafiltration tubes to remove the free dye. The sorted T helper cells were incubated with 1 × 10^8^ PKH26‐labeled exosomes for 24 h. The cells were then washed three times with PBS. The DiO dye and PBS were prepared as working solutions at a ratio of 1:100, after which 200 µL of the working solution was added to each well, incubated for 1 h at room temperature, and washed three times with PBS. Finally, 200 µL of DAPI‐containing mounting medium was added to each well of the Petri dish and imaged using a Nikon confocal microscope.

### Th17 Cells Differentiation

Human Th17 cells were differentiated from naïve CD4^+^ T cells isolated from human peripheral blood monocytes (PBMCs). Briefly, human PBMCs were prepared from the buffy coat by density gradient centrifugation using Ficoll‐Paque. Naïve CD4^+^CD45RA^+^ T cells were purified from PBMCs using a Naive T Cell Isolation Kit. According to the manufacturer's protocol of the T Cell Activation/Expansion Kit, naïve CD4^+^ T cells were seeded and cultured in a serum‐free medium. After activation, cells were cultured in the presence of the Th17‐polarizing cytokines IL‐1β (20 ng mL^−1^), IL‐6 (30 ng mL^−1^), IL‐23 (30 ng mL^−1^), TGF‐β1 (2.25 ng mL^−1^), anti‐IFN‐γ (1 µg mL^−1^), and anti‐IL‐4 (2.5 µg mL^−1^) antibodies. The cells were cultured for 7 days at 37 °C in an atmosphere of 5% CO_2_ without any media exchange.

### Th17 Cells Isolation from Bone Marrow

Human bone marrow cells were isolated by flushing the femur. Cells were washed with phosphate‐buffered saline with 2% FBS, 2 mm EDTA and stained with Anti‐Human CD4, Anti‐Human CXCR3, Anti‐Human CCR6, Anti‐Human CCR4, Anti‐Human CD161 and incubated at 37 °C for 60 min. After centrifugation, cells were resuspended in phosphate‐buffered saline with 2% fetal bovine serum, 2 mm EDTA, and then filtered through a 100 µm cell strainer. Th17 cells (CD4+CXCR3‐ CCR6+CCR4+CD161+) were sorted by fluorescence‐activated cell sorting (FACS) in SONY SH800S.

### RT‐qPCR Analysis

Total mRNA was extracted from cultured cells and muscle tissues using an RNA isolator and reverse transcribed into cDNA using the HiScript III All‐in‐one RT SuperMix Perfect for qPCR. Real‐time PCR was performed using ChamQ Universal SYBR qPCR Master Mix on a CFX96 Real‐Time System. ACTB was used to normalize the RNA content of the samples, and the 2^−ΔΔCt^ method was used to calculate relative expression. The primer sequences used were listed in the Key resources table.

### Co‐Culture of Macrophages and Th17 Cells

Fresh venous blood was drawn from healthy donors and anticoagulated with sodium citrate. The PBMCs were isolated from human blood by density gradient centrifugation using Ficoll‐Paque. For macrophage differentiation, PBMCs were cultured in RPMI‐1640 supplemented with 10% FBS, 1% penicillin‐streptomycin, and 100 ng mL^−1^ macrophage colony‐stimulating factor (M‐CSF) for 6 days. Then human Th17 cells (1 00 000 cells per well) were co‐cultured with macrophages for 3 days supplemented with or without human plasma exosomes (high BAG6 and low BAG6). The cell suspensions were pelleted, and the cell supernatants were harvested and stored at −80 °C for quantification of cytokines. SPP1 in cell supernatants was measured using ELISA according to the manufacturer's instructions.

## Conflict of Interest

The authors declare no conflict of interest.

## Author Contributions

P.Y., M.C., and M.R. contributed equally to this work. P.T., P.Y., and L.Z. conceived and designed the study. W.C. provided the clinical samples. M.C., R.C., X.H., and H.Y. prepared samples. Y.Z. and Y.Y. performed the cell sorting and experiments. Y.L. and M.Z. performed validation experiments under the supervision of R.X. M.R. performed the data analysis. M.C. and Y.L. helped with the clinical information analysis. X.H. and R.X. helped with data analysis and interpretation. P.Y., M.R., and M.C. wrote the manuscript with inputs from P.T.

## Supporting information

Supporting Information

Supplemental Table 1

Supplemental Table 2

Supplemental Table 3

Supplemental Table 4

Supplemental Table 5

## Data Availability

The data that support the findings of this study are available from the corresponding author upon reasonable request.
