# Supplementary material for: Deciphering Immune Landscape Remodeling Unravels the Underlying Mechanism for Synchronized Muscle and Bone Aging
Source: Adv Sci (Weinh). 2023 Dec 13;11(5):2304084. doi: 10.1002/advs.202304084 (PMC10837389; doi:10.1002/advs.202304084)
Supplement: Supplementary file 1 — Supporting Information [file ADVS-11-2304084-s001.pdf]

ADVANCED SCIENCE
Open Access

Supporting Information

for *Adv. Sci.*, DOI 10.1002/advs.202304084

Deciphering Immune Landscape Remodeling Unravels the Underlying Mechanism for Synchronized Muscle and Bone Aging

Pengbin Yin, Ming Chen, Man Rao, Yuan Lin, Mingming Zhang, Ren Xu, Xueda Hu, Ruijing Chen, Wei Chai, Xiang Huang, Haikuan Yu, Yao Yao, Yali Zhao, Yi Li, Licheng Zhang and Peifu Tang**

CCL2 derived from aged SMC may explained a prolonged inflammatory CCR2 signaling which damps muscle regeneration while ageing

Classical arterial, capillary, lymphatic, and venous EC phenotypes were identified (Supplementary Fig. S2a, b). An increased enrichment in genes responsive to oxidative stress (*CD36, FABP4, DUSP1,* and *APOD*) were seen in all aged EC subsets (Supplementary Fig. S2c). In addition, venous EC acquired a pro-inflammatory feature while ageing which highly express *ACKR1, DUSP23, PLVAP,* and *SELE* (Supplementary Fig. S2c). Functional enrichment also found that aged venous EC highly expressed genes in related to aging, blood vessel branching, and extracellular matrix (ECM) (Supplementary Fig. S2d). The latter two biological processes were also known for involvement in structural remodeling of the microvasculature. Smooth muscle cells (SMC) were mostly identified in muscle (Supplementary Fig. S2e). An ageing phenotype was seen in aged SMC, displayed as function enrichment in iNOS and programmed cell death and ROS (Supplementary Fig. S2f). Of note, an increase in *CCL2* expression is unique in SMC (Supplementary Fig. S2g). Prior showed an increased activation of *CCR2* pathway in muscle is associated with ageing related muscle loss [1]. Therefore, we postulated the origin of the ligand may partially derived from aged SMC-secreted *CCL2*. Thus, we use SENNIC analysis to identify the potential TFs for *CCL2*, from which *FOSL2* was identified (Supplementary Fig. S2h).

1 Blanc, R. S. *et al.* Inhibition of inflammatory CCR2 signaling promotes aged muscle regeneration and strength recovery after injury. *Nat Commun* **11**, 4167, doi:10.1038/s41467-020-17620-8 (2020).

Supporting Information

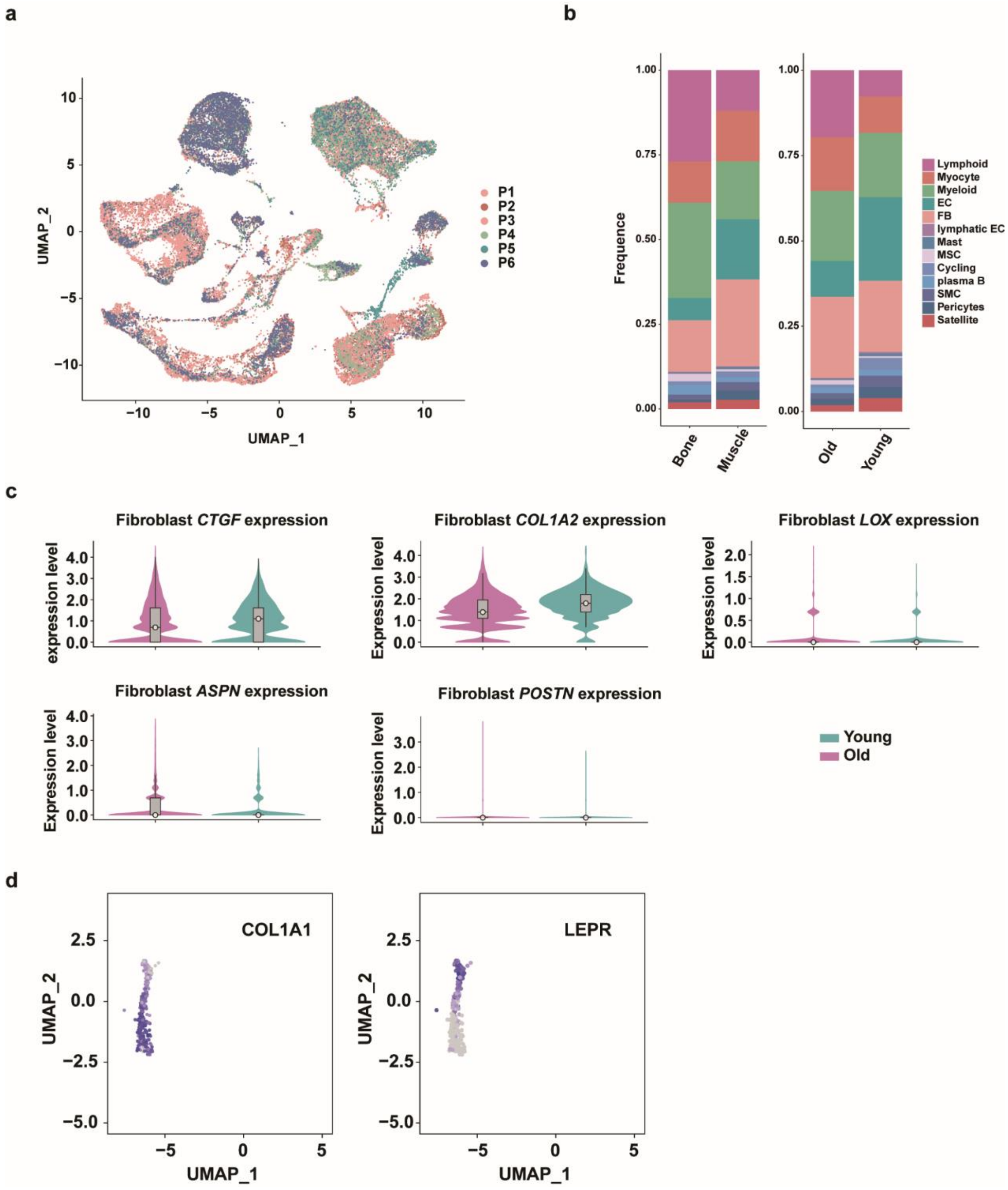

Supplementary Fig. S1. Cell subsets identification and comparison a. Cell clustering projected by UMAP plots showing major cell types in musculoskeletal tissues detected by scRNA-seq; colored by individual donor **b.** Cell-subset composition comparison in bone and muscle during aging. c. Fibrogenic gene expression between old and young individuals indicated their muscles were uninjured. d. UMAP plot showing differential expression of *LEPR* and *COL1A1* within the MSC cluster.

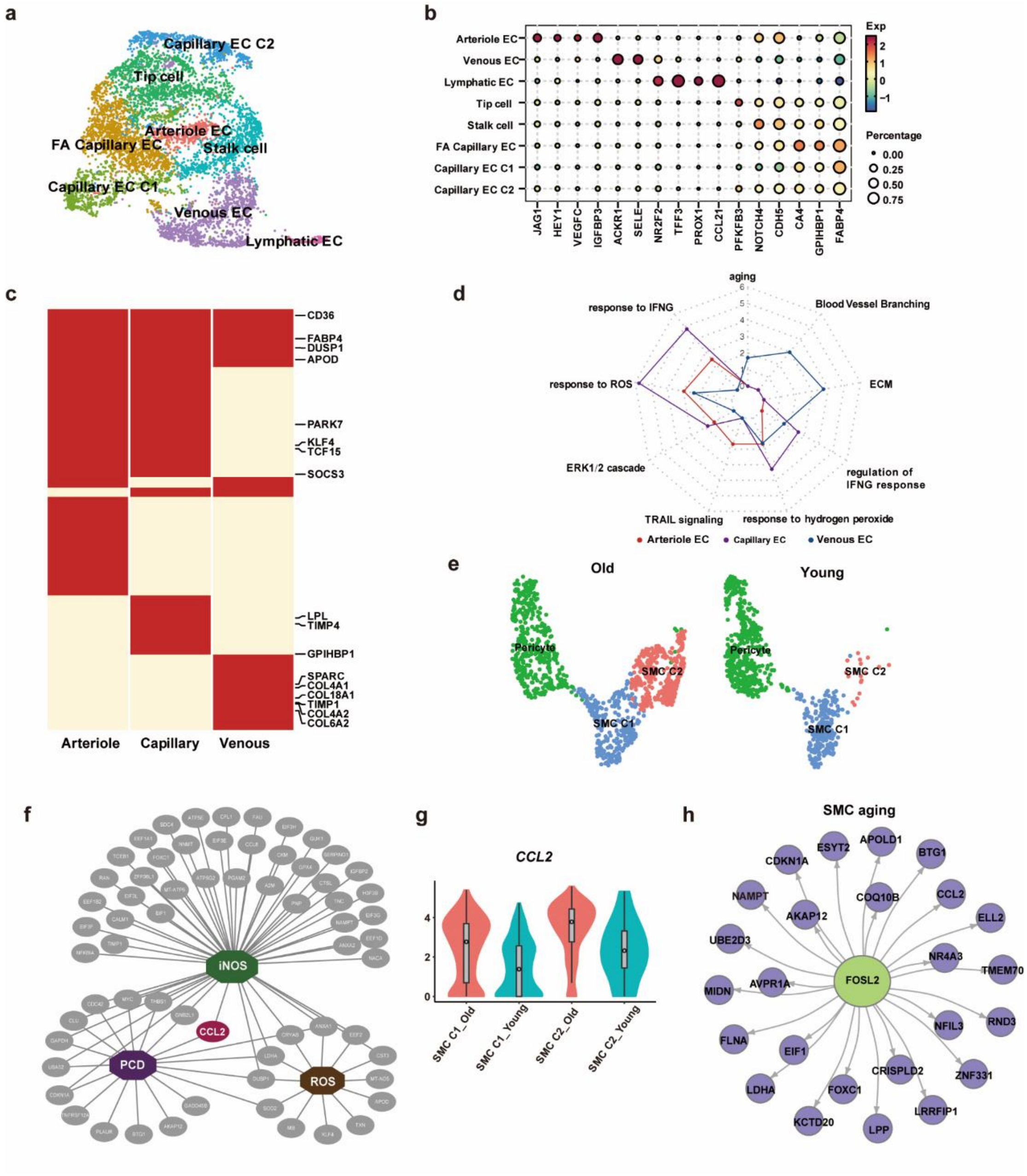

Supplementary Fig. S2. Endothelial cells (ECs) and smooth muscle cells (SMCs) in human bone and muscle tissue. a. UMAP plots showing the diversity of ECs. b. Dot plot showing distinctive gene expression in each subset. c. Heatmaps showing the distribution of upregulated genes (Red) in each arteriole, capillary, and venous EC groups in old individuals compared to young individuals. d. The functional enrichment of highly expressed genes in arteriole, capillary, and venous EC groups, visualized by a Radar plot. e. UMAP plots showed the diversity of SMCs. f. The gene expression network inferred *CCL2* as a hub target of iNOS, programmed cell death, and ROS processes. g. *CCL2* expression comparison in SMC_C1 and SMC_C2 subsets between young and old individuals, visualized by a Violin plot. h. Gene regulatory network

inferred *FOSL2* as a potential regulator of *CCL2* expression.

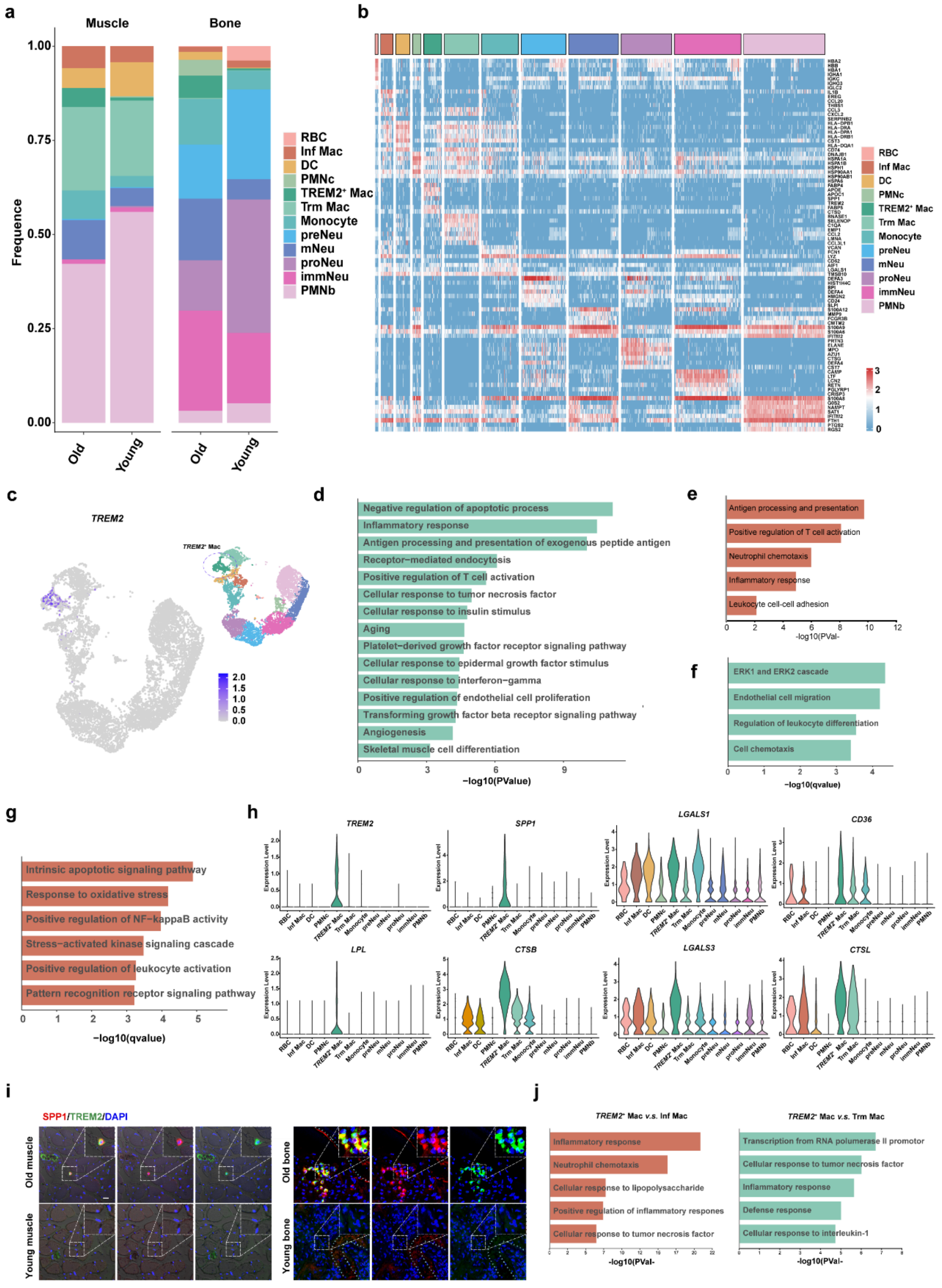

Supplementary Fig. S3. Myeloid cells in human bone and muscle tissue. a. Myeloid cell-subset composition comparison in bone and muscle during aging. b. Heatmap showing the signature expression of each cell subset. c. UMAP plot showing the unique expression of *TREM2* in *TREM2*$^{+}$ Macs d. GO enrichment of transcriptional signature genes in Trm Mac. e. GO enrichment of transcriptional signature genes in Inf Mac. f. GO enrichment of highly expressed gene in aged Trm Mac. g. GO enrichment of highly

expressed gene in aged Inf Mac. h. Violin plot of genes highly expressed in *TREM2*$^+$ Macs. i. Representative images of immunofluorescent staining for TREM2 and SPP1 in both muscle and bone from young and aged individuals. j. GO enrichment of highly expressed genes in Inf Mac and Trm Mac, as compared to *TREM2*$^+$ Mac

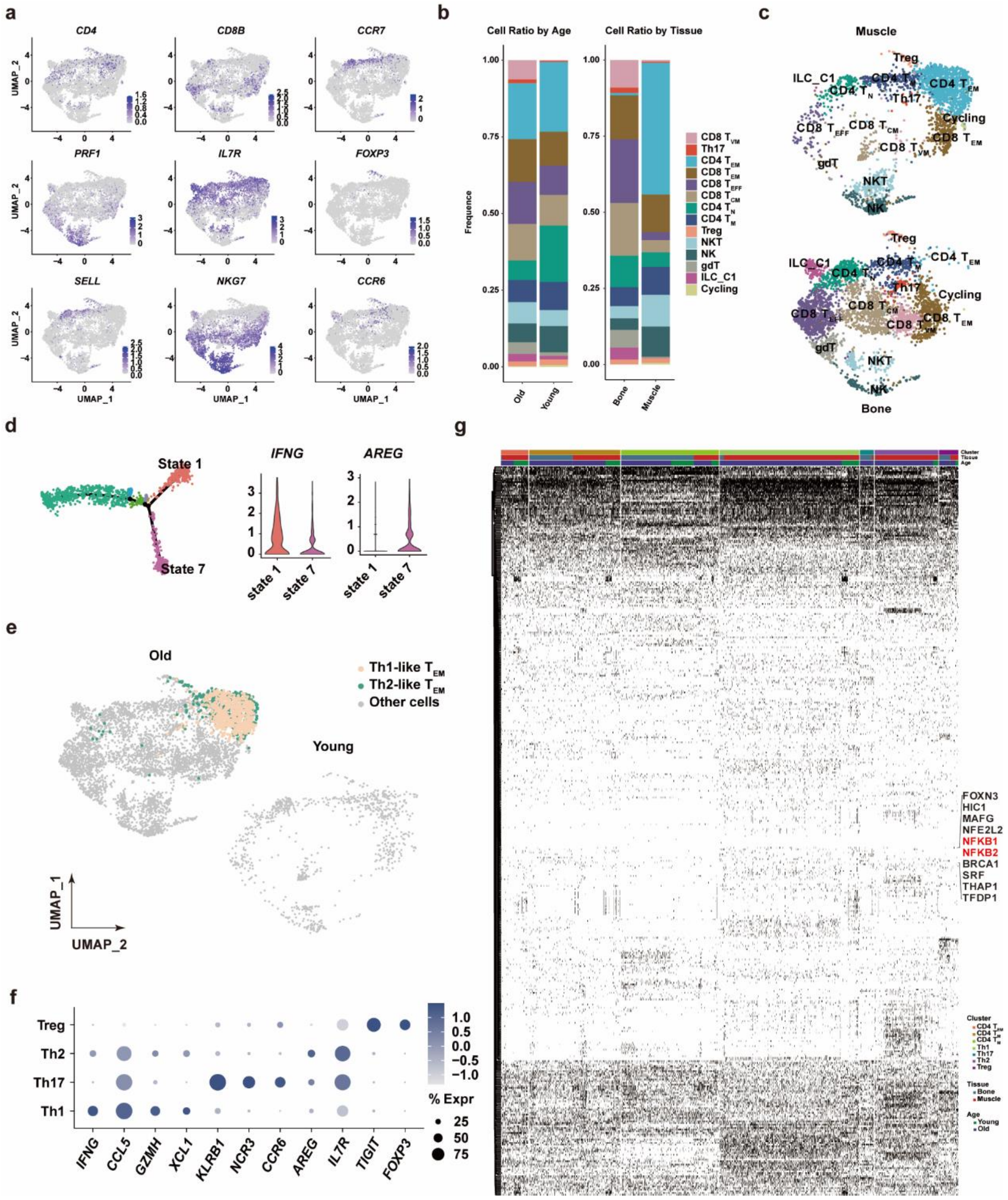

Supplementary Fig. S4. Lymphoid cells in human bone and muscle tissue. a. Canonical markers projected on a UMAP plot. b. Lymphoid cell-subset composition comparison in bone and muscle during aging. c. UMAP plots separated by tissue. d. Trajectory inference of $CD4^+$ T cell subsets by Monocle. e. UMAP plot showing the presence of Th1-like and Th2-like effector memory cells in old muscle but not in young muscle. f. Dot plot showing differentially expressed genes among Th1, Th2, Th17, and Treg. g. The overall regulon binary map of $CD4^+$ T cells via SCENIC, which was plotted and grouped by cluster, tissue, and age. Regulons of interest were highlighted.

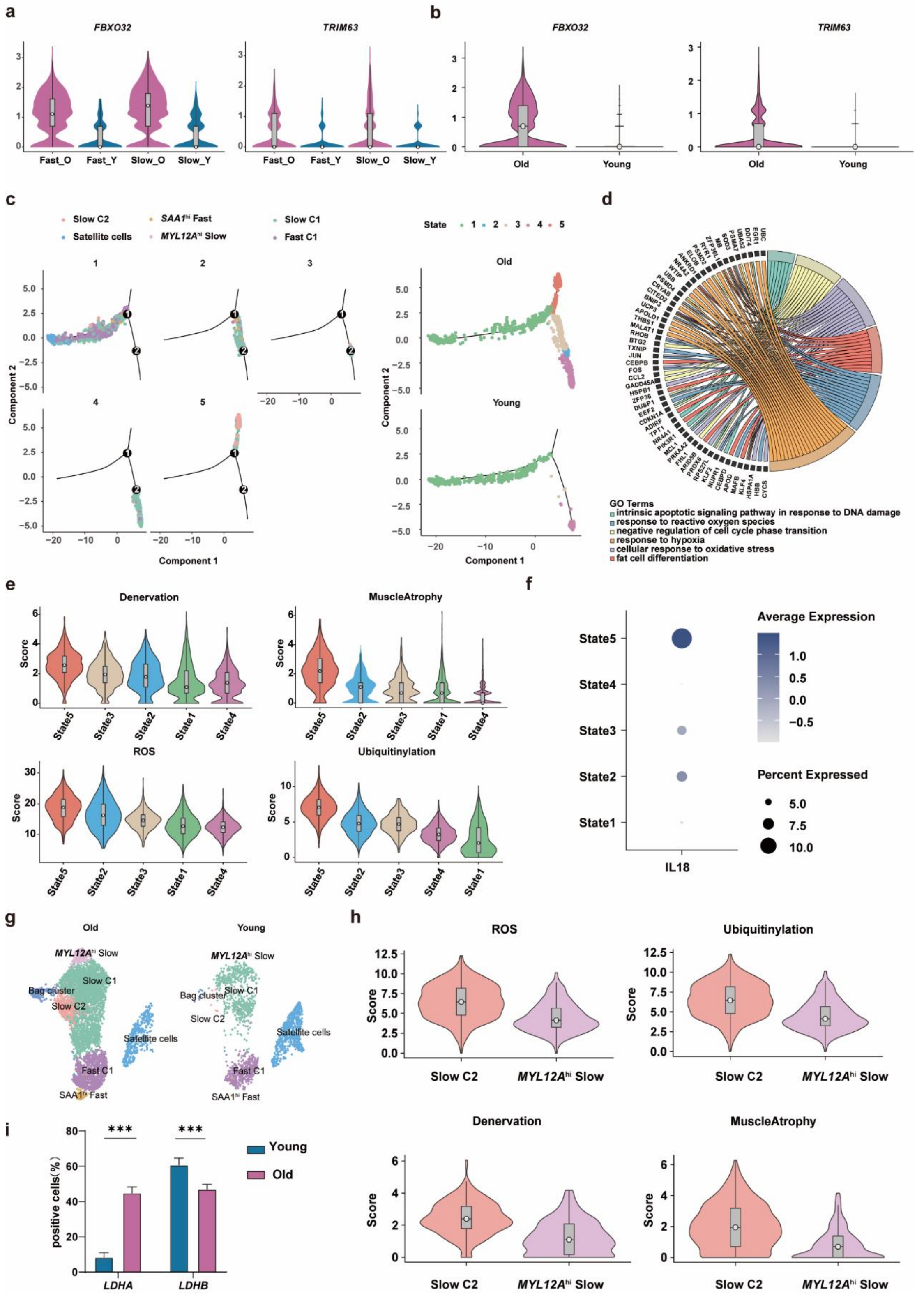

Supplementary Fig. S5. Myocytes in human bone and muscle tissue. a. Violin plot of aging hallmark genes (*TRIM63* and *FBXO32*) in fast and slow myocytes during aging. Fast_ is for Fast myocytes; Slow_ for Slow myocytes. _O is for _Old; _Y for _Young. b. *TRIM63* and *FBXO32* expression between old and young individuals. c. The trajectory of myocytes separately by State (left) and age (right). d. GO plots of highly

expressed terms/genes in State 5. e. Functional score comparison of age-related terms among different States. f. Dot plot showing high IL18 expression in State 5. g. UMAP plots of myocyte split by age. h. Functional score comparison of age-related terms between Slow C2 and *MYL12A*^{hi} Slow. i. Quantification of *LDHA* and *LDHB* positive cells in Fig. 4j.

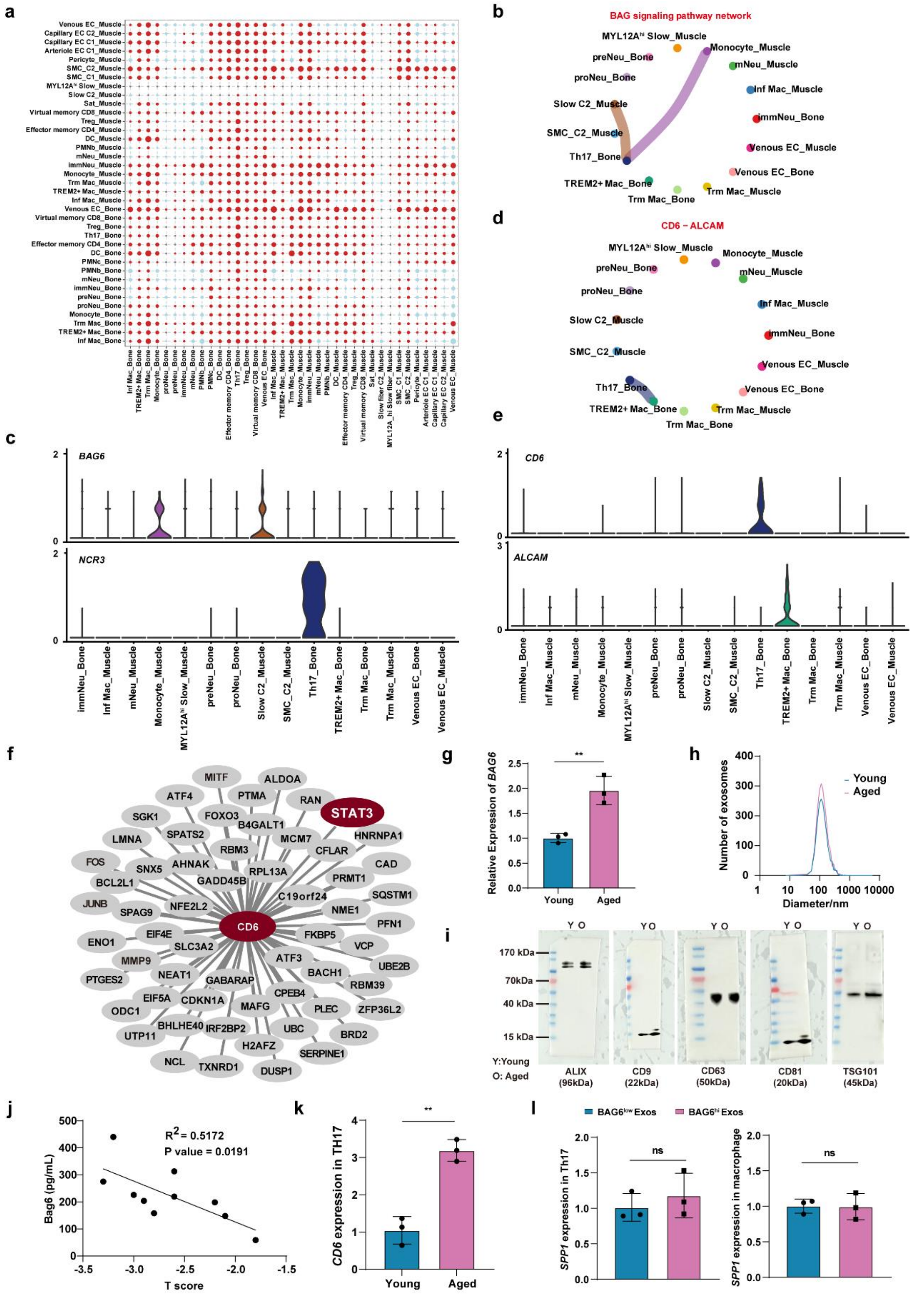

Supplementary Fig. S6. Cell-cell interaction in different subsets between human bone and muscle tissues. a. Graphic representation of altered cell-cell crosstalk in aging. Circle size indicates interaction weight changes between sender cells and receiver cells. Red accounted for increased weight in aging, whereas blue projected reduced cell-cell communication. b. The BAG signaling pathway was mainly sent by

Slow C2 and monocytes in muscle and received by Th17 in bone. c. Violin plot showing ligand-receptor pairs in BAG signaling. BAG6 was a ligand, and NCR3 was a receptor expressed by Th17. d. The CD6-ALCAM pair were uniquely found between Th17 and *TREM2*$^+$ Macs in bone. e. Violin plot showing ligand-receptor pairs in CD6 signaling. The ligand CD6 was highly expressed by Th17, and ALCAM, as its receptor, was specifically expressed by *TREM2*$^+$ Macs. f. NicheNet analysis predicted CD6 targets in *TREM2*$^+$ Macs. These results revealed that the transcription factor, *STAT3* involved in the transcription of *SPP1* (highlighted in red) were activated by CD6 stimulation. g. *BAG6* expression in the muscle from young and aged individuals, as tested by qPCR (n = 3 independent donors). h. Size distribution of exosomes derived from young and older individuals, determined by nanoparticle tracking analysis (NTA). i. Western blot images for the markers of exosomes. j. Pearson's correlation analysis between T scores and BAG6 expression levels in exosomes (n = 10). k. Intracellular CD6 expression of Th17 cells sorted from either young or old individuals, as tested by qPCR (n = 3 independent donors). l. Intracellular expression level of *SPP1* in the Th17 cells (left) and macrophages (right) after treatment with BAG6^{hi} exosomes (BAG6^{hi} Exos) or BAG6^{low} exosomes, respectively, determined by qPCR.
